# Cell division angle predicts the level of tissue mechanics that tune the amount of cerebellar folding

**DOI:** 10.1242/dev.202184

**Published:** 2024-02-13

**Authors:** Amber G. Cook, Taylor V. Bishop, Hannah R. Crowe, Daniel N. Stevens, Lauren Reine, Alexandra L. Joyner, Andrew K. Lawton

**Affiliations:** ^1^ Department of Biological Sciences, Mississippi State University, MS 39762, USA; ^2^ Developmental Biology Program, Sloan Kettering Institute, NY 10065, USA

**Keywords:** Cerebellum, Brain folding, Cell division angle, Tissue mechanics

## Abstract

Modeling has led to proposals that the amount of neural tissue folding is set by the level of differential expansion between tissue layers and that the wavelength is set by the thickness of the outer layer. Here, we used inbred mouse strains with distinct amounts of cerebellar folding to investigate these predictions. We identified a distinct critical period during which the folding amount diverges between the two strains. In this period, regional changes in the level of differential expansion between the external granule layer (EGL) and underlying core correlate with the folding amount in each strain. Additionally, the thickness of the EGL varies regionally during the critical period alongside corresponding changes in wavelength. The number of SHH-expressing Purkinje cells predicts the folding amount, but the proliferation rate in the EGL is the same between the strains. However, regional changes in the cell division angle within the EGL predicts both the tangential expansion and the thickness of the EGL. Cell division angle is likely a tunable mechanism whereby both the level of differential expansion along the perimeter and the thickness of the EGL are regionally tuned to set the amount and wavelength of folding.

## Teaser

**Highlighted Article:** Comparison of two mouse strains reveals that cell division angle within the external granule cell layer of the cerebellum varies regionally and correlates with tissue mechanics that could set the amount and wavelength of folding.

## INTRODUCTION

The human cortex and cerebellum are formed into complex folded shapes that provide space for the substantial neural circuits and a gross 3D compartmentalization of functional circuitry within each structure. Changes to the amount of folding in the brain are associated with intellectual disabilities, epilepsies and other health issues (Flotats-Bastardas et al., 2012; Leventer et al., 2010; Raymond et al., 1995). Therefore, determining how the degree of folding is set during development is crucial for understanding the functional role of the 3D partitioning of circuits in the gyri of the cortex and the lobules of the cerebellum.

Little is known about how the geometry of a tissue (the shape of a tissue and the relative arrangement of its parts) and its mechanics work together to set the proper amount of folding during development. The murine cerebellum has eight to ten folds aligned in the anterior-posterior axis in the medial region (vermis). This simple arrangement allows for a precise analysis, by examining sagittal sections, of the geometric and mechanical parameters that underlie the degree of folding that occurs during development.

Folded neural tissues have different properties than other tissues that undergo folding-like events during development (Nelson, 2016; Shyer et al., 2013; Varner and Nelson, 2014). In the developing brain, cells are arranged, not in simple epithelial/mesoderm layers, but in dynamic and thick laminar structures. The cerebellum is further characterized by the rapid expansion of a temporary external granule layer (EGL) that covers its entire outer surface. The granule cell progenitors (GCPs) within the outer EGL (oEGL) are proliferative and motile, whereas those in the inner EGL (iEGL) differentiate by extending their processes in a polarized manner along the medio-lateral axis before migrating radially into the forming inner granule layer. The proliferation of GCPs is responsible for most of the cerebellar volume growth and for directing growth primarily in the anterior-posterior axis, the axis of folding (Joyner and Bayin, 2022; Lawton et al., 2023, 2019; Legué et al., 2016, 2015; Leto et al., 2016). Within the oEGL, GCP division is symmetric, either producing two progenitors or, at the final division, producing two post-mitotic granule cells (Espinosa and Luo, 2008; Legué et al., 2016; Zong et al., 2005).

Previously, we demonstrated that cerebellar folding is initiated when the ratio of growth between the EGL and the underlying core diverges enough to create a differential expansion between the layers, with the EGL growing faster (Lawton et al., 2019). This differential-expansion process provides the driving force of initial cerebellar folding. Furthermore, we showed how the initial geometry of the unfolded tissue sets the ratios of growth required to create the differential expansion that will result in folding. These tissue mechanics, along with tissue tension and the fluid-like behavior of the EGL cells, are thought to fold and shape the cerebellum during initial development (Engstrom et al., 2018).

One prediction concerning how the folding amount is determined is that the number of folds is set by the level of differential expansion between the outer layer, the EGL, and the underlying cerebellar core. A second is that the wavelength of folding (the direct distance between the base of each pair of fissures) is defined by the thickness of the EGL (Engstrom et al., 2018; Lawton et al., 2019; Tallinen et al., 2016, 2014). The folding amount in the adult cerebral cortex across many species correlates with the thickness and surface area of the cerebral cortex, which is considered its corresponding outer layer during folding (Mota and Herculano-Houzel, 2015). Further, the variation of folding within individual cerebral cortices also follows this same relationship (Wang et al., 2019). These results led us to investigate whether the amount of cerebellar folding is set during development by adjusting both the amount of differential expansion between the EGL and core and the thickness of the EGL.

Here, we used a gene-agnostic approach to assess the tissue mechanics and tissue geometries predicted to regulate the amount of folding during development. We utilized two inbred strains of mice with small, but robust differences in the final number of vermis folds. We report that the level of differential expansion between the EGL and core during development is regionally regulated and correlates with the degree of folding, as predicted. Further, we demonstrate that the thickness of the EGL also regionally varies and is consistent with the observed corresponding wavelengths. Differences in the amount of tangential expansion of the EGL in each lobule and their initial geometry appear to work together to regionally influence the level of differential expansion between the EGL and core. We also uncovered that regional differences in the cell-division angle within the EGL predicts the tangential expansion of the EGL and its thickness. We propose that cell-division angle is a tunable mechanism whereby both the level of differential expansion of two layers and the thickness of one (the EGL) are precisely tuned and thus can set the amount of folding in the cerebellum.

## RESULTS

### Inbred mouse strains have regionally distinct levels of folding

**Fig. 1.**
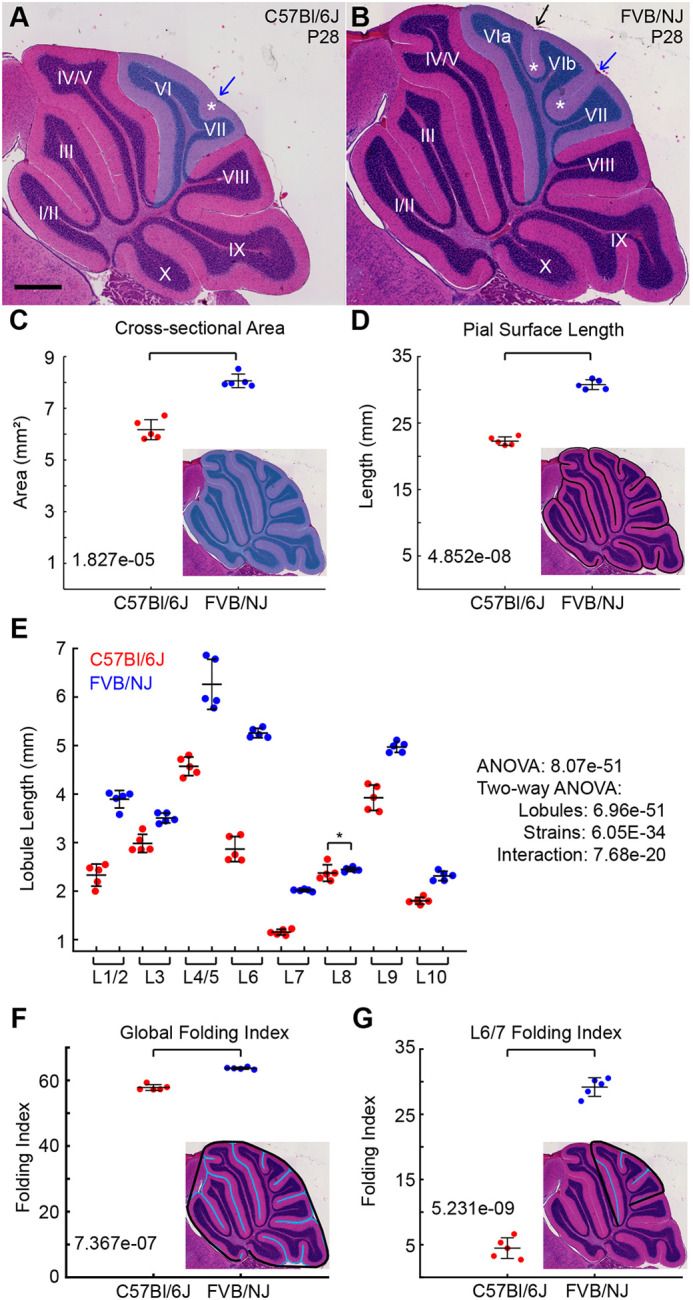
**Mouse strains have regionally varied levels of cerebellar folding.** (A,B) H&E-stained sagittal midline sections of P28 C57Bl/6J (A) and FVB/NJ (B) cerebella. Shaded region: lobules 6 and 7. Black arrow indicates superior-declive fissure (SDF), blue arrow indicates intercrural fissure (IF) and asterisks indicate anchoring centers (ACs). Scale bar: 500 µm. (C) Cross-sectional sagittal cerebellar area (as indicated by the shaded area in the inset) of each strain. (D) Pial surface length (as indicated by the black line in the inset) of each strain. (E) Lobule length of each strain. All lobule lengths are smaller in C57Bl/6J except for L8. (F,G) Global folding index (F) and regional folding index of L6/7 (G) of both strains. In insets, cyan line indicates the pial surface length and black line indicates the positive curvature of pial surface length. *n*=5 per strain for all analyses. *P*-values are shown for each analysis.

We used the C57Bl/6J and FVB/NJ inbred strains of mice because they have a robust difference in the amount of cerebellar foliation (Sillitoe and Joyner, 2007). Here, we considered postnatal day (P) 28 an ‘adult’ stage as the cerebellum has reached its final size and folding amount. In both strains, most cerebella have normal cytoarchitectural layers (Fig. 1A,B, Fig. S1A-C) and are both considered ‘wild type’. A subset of C57Bl/6J mice, however, have heterotopia, which disturbs the pattern of folding in the posterior cerebellum (Fig. S1B) (Van Dine et al., 2015). Individuals or lobules showing malformation were excluded from this study.

We found that the C57Bl/6J cerebellum is significantly smaller than the FVB/NJ, in midsagittal sectional area, pial surface length, and the positive curvature of the pial surface length, a measure of the exposed surface length (Fig. 1C,D, Fig. S2A). Globally, the C57Bl/6J cerebellum was about ∼72-75% the size of FVB/NJ in surface length and section area. Dividing the cerebellum into the lobules that are fully separated in both genotypes revealed that the size decrease in C57Bl/6J was not uniform, but regionally adjusted across the cerebellum. Lobules (L) 6 and L7 were much shorter whereas L8 was similar in size compared with FVB/NJ (Fig. 1E, Fig. S2B). We next measured the lobule lengths as a percentage of the total length of the cerebellum. Although several lobules comprised the same proportion of the total cerebellum in C57Bl/6J and FVB/NJ (L4/5, L7, L9, L10), some were proportionally larger (L3, L8) or smaller (L1/2, L6) in C57Bl/6J compared with FVB/NJ (Fig. S2C).

We measured the folding index (see Materials and Methods) to quantify the amount of folding (Lawton et al., 2019). The C57Bl/6J cerebellum was ∼10% less folded than that of FVB/NJ (Fig. 1F). However, the regional folding of the L6/7 region was 85% less in C57Bl/6J than FVB/NJ (Fig. 1G). In this region, the C57Bl/6J lacks the superior-declive fissure (SDF) separating sub-lobules 6a and 6b and the depth of the intercural fissure (IF) separating sub L6b from L7 was much smaller than in FVB/NJ (black and blue arrows, respectively in Fig. 1A,B). The folding index was thus regionally affected. The folding index of the anterior region of the cerebellum (L1-5) was ∼8% smaller and the posterior (L8-10) was ∼13% smaller in C57Bl/6J compared with FVB/NJ (Fig. S2D,E). The robust folding difference in the L6/7 region in these two strains provides a tractable system in which to determine whether differential expansion and EGL thickness could be responsible for regulating folding amount during development.

### The EGL/core growth ratios diverge at the onset of folding differences

Differential expansion can emerge between tissue layers that expand at different rates (Hannezo et al., 2012; Nelson, 2016; Shyer et al., 2013; Wiggs et al., 1997). Increasing or decreasing the level of differential expansion is predicted to change the amount of folding (Tallinen et al., 2014). Although the adult cerebellum has several layers and has been treated as a tri-layer during folding (Lejeune et al., 2016), the developing cerebellum is not separated into all the distinct cytological layers seen in the adult during the early stages of folding. The full suite of cellular layers progressively become distinct as folding continues after birth in mouse. The first folds arise embryonically when the only distinct layers are the EGL and the underlying core, which includes the Purkinje cells, interneurons and glia. Therefore, we have treated the mouse developing cerebellum as functionally behaving as a two-layer system: the outer and highly proliferative EGL and the inner core, which expands through addition of cells from several sources.

**Fig. 2.**
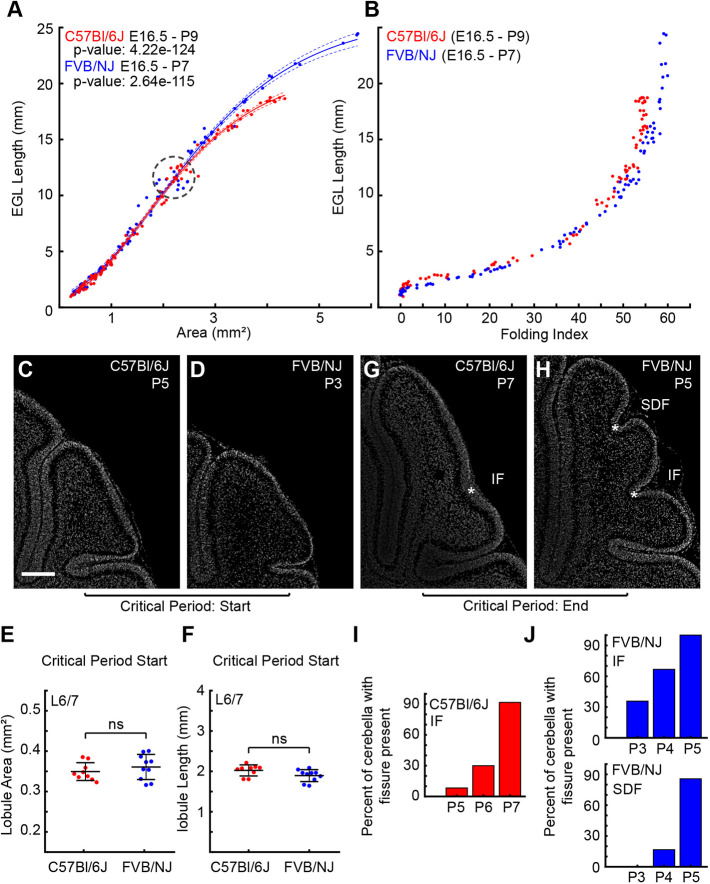
**Folding differences arise during the postnatal period.** (A) Growth ratios of the EGL length and cerebellar area from E16.5 to the formation of the final fissures. Lines represent Gompertz fitting with confidence intervals. lease explain the dashed circle in Fig. 2A. FVB/NJ: *n*=96 C57Bl/6J: *n*=94. A subset of FVB/N data below 1 mm^2^ was previously published (Lawton et al., 2019). (B) Folding index. (C,D) Example images of the L6/7 region at the start of the critical period. Scale bar: 200 µm. (E,F) L6/7 region has the same lobule area and length between strains at the start of the critical period. C57Bl/6J: *n*=9; FVB/NJ: *n*=10. ns, not significant. (G,H) Example images of the L6/7 region at the end of the critical period showing acquisition of the intercrural (IF) and the superior-declive (SDF) fissures. Asterisk indicates ACs at the base of each fissure. (I,J) Percentage of cerebella showing AC acquisition. C57Bl/6J: P5: 1/12, P6: 3/10, P7: 11/12; FVB/NJ SDF: P3: 5/14, P4: 8/12, P5: 14/14; FVB/NJ IF: P3: 0/14, P4: 2/12, P5: 12/14.

We measured the increase in both midsagittal EGL length and cerebellar sectional area from embryonic day (E) 16.5 through the stage when the final anchoring centers (ACs), the bases of the fissures, form (Fig. 2A). This allowed an unbiased comparison of the growth of the strains, controlling for any global differences in the rates of development of the different strains, actual ages of the various litters due to mating time variation, and growth effects from litter size. The global growth data were found to be well fitted by a basic Gompertz function (Tjørve and Tjørve, 2017). This function was chosen for its minimal variables, its historic use in modeling biological growth, and how well it models the complex data. The non-linear regression analyses for the individual strains showed a better fit with more uniform residuals and distinct parameters for each strain than with a combined fit (Fig. S3A-E). Additionally, we examined whether the developing cerebella of the two strains shared the same universal scaling relationship reported for the cerebral cortex whereby the exposed surface scales with the cortical thickness and surface area (Mota and Herculano-Houzel, 2015). We found that the growth profiles of the two strains, although similar, did not have the same universal relationship, but rather they diverged as development progressed. Further, the residuals were not uniform, or evenly dispersed, suggesting a poor goodness-of-fit under this model (Fig. S3F-J).

We next focused on the EGL length/core area growth ratio and found that both C57Bl/6J and FVB/NJ had the same ratio of growth between the EGL expansion and the area expansion until they reached a sagittal area of over ∼2 mm^2^ (Fig. 2A). Correspondingly, during this period the folding indices for each strain mostly overlapped (Fig. 2B). This result indicates that the early growth ratios and tissue geometries are likely creating similar amounts of EGL/core differential expansion that lead to similar amounts of initial folding in the two strains. Therefore, mechanical differences likely arise after the cerebella reach this critical point (∼2 mm^2^ sagittal area) to account for the greater amount of folding in the adult FVB/NJ cerebellum compared with C57Bl/6J.

Interestingly, the C57Bl/6J and FVB/NJ cerebella reach the critical size at different ages, approximately P5 and P3, respectively. This finding demonstrates that FVB/NJ cerebella grow more rapidly than C57Bl/6J, to reach the same size roughly 2 days earlier. However, the crucial factor for determining the final amount of folding is not the global speed of growth but likely the level of differential expansion that emerges from the growth ratio between the EGL and the core area after the critical point.

As the cerebella expanded beyond an area of ∼2 mm^2^, the FVB/NJ EGL continued to expand at the same ratio whereas the ratio of EGL/core area expansion in the C57Bl/6J was reduced (Fig. 2A). Once the growth ratios diverged between the strains, the amount of folding in the cerebellum also diverged (Fig. 2B). Furthermore, in both strains, when the cerebella were ∼2 mm^2^, the L6/7 region was unfolded and was the same size, as determined by both EGL length and lobule area measurements (Fig. 2C-F). However, as the global growth ratios diverged, the difference in the number of folds in the L6/7 region (one in C57Bl/6J and two in FVB/NJ) become apparent (Fig. 2G,H). We identified a critical period of development for the L6/7 region when the SDF and IF fissures appear and the folding amount between the strains diverges. This period coincides with an age of ∼P5-P7 for C57Bl/6J and ∼P3-P5 for FVB/NJ (Fig. 2I,J).

### Cerebellar strains have similar geometry and EGL/core growth ratios at folding initiation

Previously, we provided evidence that cerebellar folding emerges without a molecular prepattern determining the location of the folds (Lawton et al., 2019). Still, it could be that differences in the initial size or geometry of the cerebella of each strain could give rise to the differences in the amount of folding that become apparent later in development. However, we found that at E16.5 the strains have the same midsagittal cerebellar area, EGL length, and geometric relationship between their length and area (Fig. S4A-C). We also compared the growth ratio of the EGL and core at the initiation of folding (from E16.5 until the cerebella reach a sagittal area of 1 mm^2^, corresponding to ∼P0 for both strains). Multiple linear regression analysis showed that there was no difference between the EGL/core growth ratios (Fig. S4D-G). Together, these results provide evidence that the difference in folding amount is not pre-figured by differences in the geometry of the cerebellar anlagen or embryonic growth ratios between the two strains.

### The EGL/core growth ratio is greater in L6/7 of FVB/NJ compared with C57Bl/6J

**Fig. 3.**
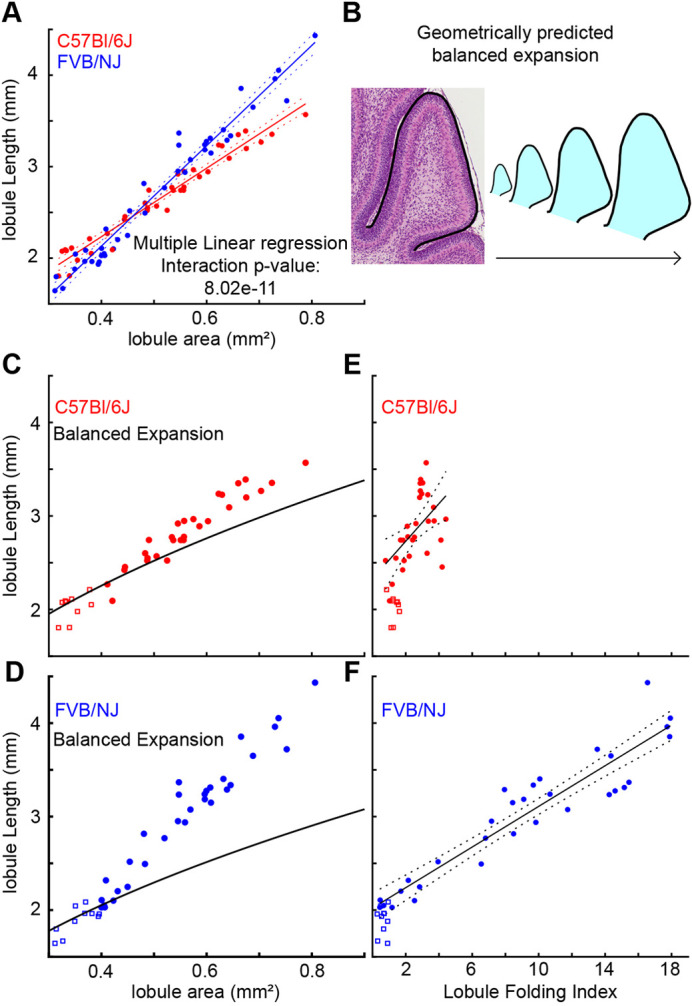
**The level of differential expansion sets the folding amount.** (A) Growth ratios of the L6/7 region during the critical period. (B) Balanced expansion curve created by isometric scaling of lobule regions from the start of the critical period (squares in C-F). Solid lines: linear regression fit. Dotted lines: 95% confidence intervals. (C) The C57Bl/6J growth ratio remains near the balanced growth curve. (D) The FVB/NJ growth ratio exceeds the balanced growth curve r. (E) The C57Bl/6J L6/7 folding index shows limited folding increase. (F) The FVB/NJ L6/7 folding index reveals an increase in folding. For all analyses, C57Bl/6J *n*=38; FVB/NJ *n*=41.

We next examined whether the EGL/core growth ratio was different between the strains within the L6/7 region where there is a clear difference in the level of folding. Given that the ACs, the base of fissures, maintain fixed positions within the cerebella and that growth is constrained within each lobule, it is possible to determine regional levels of expansion and folding by treating the ACs as boundaries (Lawton et al., 2019; Legué et al., 2015; Sudarov and Joyner, 2007; Szulc et al., 2015). We measured the expansion rate of L6/7 starting at the beginning of the critical period (roughly P3 for FVB/NJ and P5 for C57Bl/6J) when the area of L6/7 was at least 0.3 mm^2^, until the area was no more than 0.8 mm^2^ (roughly P6 for FVB/NJ and P8 for C57Bl/6J). Multiple linear regression analysis showed that the growth ratio (EGL length/cortex area) of L6/7 of C57Bl/6J cerebella is smaller compared with FVB/NJ (Fig. 3A).

### The level of EGL/core differential expansion is regionally distinct and correlates with the cerebellar folding amount

We next examined whether the growth ratios (EGL/core) are regionally regulated within each strain. We first measured the growth ratio of L4/5 and L8 from the same individuals used for the measurements for L6/7. In C57Bl/6J, both L4/5 and L8 had smaller EGL/core growth ratios compared with their FVB/NJ counterparts (Fig. S5A,B). Multiple linear regression analysis showed that within C57Bl/6J there was no difference between the EGL/core growth ratios of L4/5, L6/7 or L8, whereas within FVB/NJ L6/7 was statistically different from L4/5. However, the change was minute and may not be biologically relevant (Fig. S5C-E).

A differential expansion between layers emerges when the EGL/core growth ratio exceeds that of balanced expansion (the ratio of growth of each layer needed to maintain isometric scaling of a given 2D geometry), which is defined by the tissue geometry (Fig. S5F). To determine whether the measured difference in growth ratios between the strains constitutes a change in the level of EGL/core differential expansion, we constructed balanced-expansion curves from specific lobule regions by isometrically scaling the median lobule region from the start of the critical period (L6/7 regions with an area of 0.3-0.4 mm^2^; Fig. 3A,B). If the growth ratio is maintained along the balanced-expansion curve, then the growth is balanced for the geometry and no folding will occur. However, a ratio of growth where the slope exceeds the curve indicates the presence of differential expansion with the EGL growing faster than the core and predicts folding.

We found that L6/7 in C57Bl/6J has less EGL/core differential expansion than L6/7 in FVB/NJ throughout the critical period (Fig. 3C,D). The C57Bl/6J strain remained centered around the balanced growth line for longer, and when the rate of EGL expansion increased above the curve it remained closer to the curve compared with FVB/NJ. The difference in the level of EGL/core differential expansion was also visualized in the residuals of fitting the data to the predictive balanced-expansion curve (Fig. S5G). Correspondingly, both the timing of folding and amount of folding followed the onset and degree of EGL/core differential expansion in both strains (Fig. 3E,F).

Although the sizes of L6/7 were the same between the strains at the start of the critical period (Fig. 2E,F), the predictive balanced-expansion curves showed that the C57Bl/6J cerebellum requires a higher level of length expansion to produce a balanced expansion than that required in FVB/NJ (Fig. 3C,E). This indicates that there is a difference in the geometry of L6/7 between the strains. Indeed, plotting the lobule length against the area revealed that L6/7 in C57Bl/6J has a lower area-to-length ratio than FVB/NJ (Fig. S5H,I). This shift in the ratio in C57Bl/6J results in a requirement for a steeper EGL/core growth ratio to induce the same level of differential expansion as in FVB/NJ. Therefore, both the EGL/core growth ratios and the tissue geometry of L6/7 control the amount of folding in L6/7.

Within FVB/NJ, the three neighboring lobule regions, L4/5, L6/7 and L8, have very similar EGL/core growth ratios (Fig. S5C). However, each lobule regions achieves different folding amounts during the critical period, with L8 remaining unfolded, L4/5 folding once, and L6/7 folding twice. Therefore, we postulated that the distinct geometry of each lobule region must be defining unique balanced EGL/core expansion ratios that must be overcome for the individual lobule regions to fold. We focused on L8 because of its simpler shape. In both strains, L8 is largely constrained by the surrounding lobule regions. As a result, its width does not increase as the lobule grows. This non-isometric expansion can be compared with a rectangle that has a fixed width and a growing length (Fig. S6A,B). For this type of growth, the predicted balanced-expansion profile takes a linear form (Fig. S6C). We found that in both strains the EGL/core growth ratio of L8 closely approximates the geometrically determined balanced-expansion profile during the critical period, and, as predicted, produces no folding (Fig. S6D,E). Therefore, although the EGL/core growth ratios of L4/5, L6/7 and L8 are similar within each strain, the resulting level of differential expansion is regionally determined by the distinct geometries of the individual lobule regions. Lastly, the folding index of L4/5 in C57Bl/6J had a small increase of the folding index at the end of the critical period compared with at the start of the critical period. This increase resulted from the bent shape of the lobule and not the onset of new fissures (Fig. S6F,G).

### The EGL thickness varies regionally between strains

**Fig. 4.**
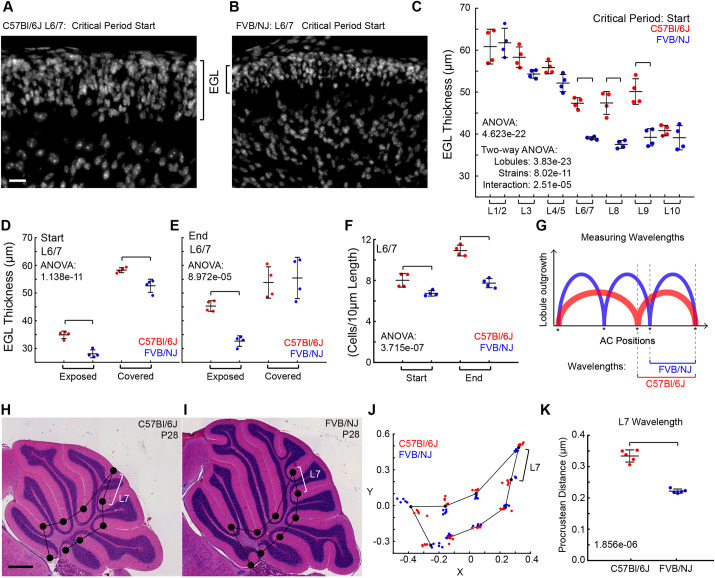
**The EGL thickness correlates with the wavelength.** (A,B) EGL of the L6/7 region. Scale bar: 20 µm. (C) EGL thickness is regionally regulated within and between the strains. (D,E) Exposed versus covered EGL thickness in L6/7 during the critical period. (F) Number of cells per 10 µm of L6/7 EGL length. (C-F) *n*=4 per strain. (G) Schematic of wavelength measurements. (H,I) Example cerebella with placement of the landmarks at the conserved ACs. White brackets indicate L7 wavelength. Scale bar: 500 µm. (J) Landmark-based procrustean analysis. *n*=5 per strain. Bracket indicates L7 wavelength. (K) Wavelength of L7 is increased in C57Bl/6J cerebella (*n*=5 per strain).

In bi-layer models of cerebral cortex folding, the thickness of the outer layer has been predicted to regulate the wavelength of the resulting folds with thicker layers predicted to produce greater wavelengths between folds (Tallinen et al., 2014). To determine whether the thickness of the EGL during the formation of the ACs could control the folding wavelength, we measured the thickness of the EGL during the critical period in each strain when the first fissure that subdivides L6/7 appeared (P5, P7 for C57Bl/6J and P3, P5 for FVB/NJ). The EGL thickness was found to vary between some lobule regions in both strains (Fig. 4A-C, Fig. S7A). Furthermore, at the start of the critical period, prior to the formation of the AC that separates L6 and L7, the EGL of L6/7, L8 and L9 was thicker in C57Bl/6J than in FVB/NJ. At the end of the critical period, the thickness of the EGL in each of L6, L8 and L9 was the same in the two strains, whereas the EGL of L7 in C57Bl/6J was significantly thicker compared with FVB/NJ (Fig. S7A). At the end of the critical period, the EGL thickness of L6 and L7 combined (L6/7) was not statistically different between the strains owing to the small proportion of L7 (Fig. S7A).

We next examined whether the EGL thickness varied within the regions of L6/7 that are exposed (where the fissures will appear) and those that are covered (adjacent to other lobules) (white and cyan, respectively in Fig. S7B). At the start of the critical period, the EGL of the exposed region was thinner than the covered region in both strains. However, the difference between the strains was most pronounced in the exposed region, with the C57Bl/6J having a thicker EGL than FVB/NJ at the start and end of the critical period. By the end of the critical period, the EGL in the covered regions showed no difference in thickness between the strains (Fig. 4D,E). As another way to measure the thickness of the exposed surface of L6/7, we quantified the number of cells in the oEGL and iEGL as a ratio of the exposed EGL surface length. The C57Bl/6J cerebella had more cells per exposed EGL length in L6/7 at both time points than in FVB/NJ (Fig. 4F), and the density of the EGL between the strains was the same (Fig. S7C).

### Final wavelength of folding in L6/7 can be predicted by EGL thickness

To determine whether the thicker EGL in some lobules of C57Bl/6J results in an increased folding wavelength, we measured the direct distance between the ACs shared by both strains at P28 (Fig. 4G-I). AC largely hold their positions in space and therefore retain the spatial information of the EGL surface from the period when they were placed (Sudarov and Joyner, 2007; Szulc et al., 2015). Further, each AC has a robustly stereotyped timing of appearance (Legué et al., 2015). Therefore, the thickness of the EGL at the time each AC forms should contribute to the final wavelength of the enclosed lobule. Given that the entire C57Bl/6J cerebellum is only ∼72-75% the size of FVB/NJ, we used a landmark-based procrustean analysis to correct for the global size difference (Fig. 4J).

Within each strain, each of the ACs showed tight clustering with minimal variation (Fig. 4J, Fig. S7D-F). Using the positions of the ACs retained in both strains, we measured the wavelength of each lobule as the direct distance between the two ACs on either side of each lobule region. The wavelength of L7 was higher in C57Bl/6J compared with FVB/NJ, as predicted, whereas the wavelengths of L1/2 and L3 were lower in C57Bl/6J (Fig. 4K, Fig. S7G). The remaining wavelengths were similar between the strains, even L8 and L9 where the EGL thickness was greater at the start of the critical period in C57Bl/6J compared with FVB/NJ. However, only the anterior AC enclosing L7 was generated during the critical period when the EGL thickness was measured to be higher in C57Bl/6. The ACs setting the boundaries of the other lobules are formed prior to this critical period when EGL thickness was not measured.

We also measured the folding wavelength in absolute distance, not correcting for size differences, and observed the same pattern of wavelengths across the cerebella as with the landmark-based procrustean analysis (Fig. S7H). All the measured ACs, save the one marking the anterior boundary of L7, were in place prior to the critical period, before any significant differences in the growth ratios of the cerebellum developed (Fig. 2A) and were maintained in position during development.

A similar lobule wavelength between strains predicts that, at the time the ACs formed, the EGL in that region had a similar thickness in the two strains. Therefore, we chose to measure the EGL thickness of L8 as its wavelength was unchanged between the strains. The secondary fissure, emerging embryonically (∼E18), marks the posterior boundary of L8. The prepyramidal fissure, marking its anterior limit, is already established in the undivided L6/7/8 region at P1 in FVB/NJ and P2 in C57Bl/6J. Therefore, we measured the EGL thickness of L6/7/8 1 day prior (P0 and P1 in FVB/NJ and C57Bl/6J, respectively). The EGL thickness was similar between the strains, as predicted, although there was a small statistically significant difference suggesting that the EGL in FVB/NJ is ∼2 µm thicker than in C57Bl/6J (Fig. S7I).

### Strains have different densities of Purkinje cells at the completion of folding

Because the level of EGL/core differential expansion and EGL thickness are different between the strains and regionally regulated within the cerebellum, we next sought to determine whether cellular mechanism could account for these differences. We first investigated the cellular players that drive expansion of the EGL and the cerebellum. Purkinje cells provide the mitogen that drives the proliferation of GCPs within the EGL (Lawton et al., 2023). Without GCP expansion the folding is severely diminished (Corrales et al., 2006). Purkinje cells also play an important role in scaling other cell populations (interneurons and astrocytes) in the cerebellar cortex to form the appropriate number of cellular partners for functional circuits (Joyner and Bayin, 2022; Willett et al., 2019). Therefore, we investigated whether the density of Purkinje cells was different between the strains in a way that could change the level of EGL expansion and, therefore, the growth ratio, differential expansion, and folding.

We first determined the total number and density of Purkinje cells per midline section in each lobule at P28 (Fig. S8A-E). The lobules have distinct numbers of Purkinje cells in each strain; L1/2 and L6 have fewer Purkinje cells in C57Bl/6J than in FVB/NJ and this accounts for slightly fewer Purkinje cells overall in C57Bl/6J. However, FVB/NJ was found to have a lower density of Purkinje cells (number/length) overall than C57Bl/6J at P28. Several lobule regions (L4/5, L6, L9 and L10) account for the lower density in FVB/NJ than C57Bl/6J. Thus, in regions where the number of Purkinje cells are the same, C57Bl/6J cerebella expand less per Purkinje cell than do FVB/NJ cerebella. Additionally, in the L6 region, where folding is most different between the strains, there were also fewer Purkinje cells in C57Bl/6J.

### During the critical period for L6/7, FVB/NJ has a higher density of Purkinje cells than C57Bl/6J

**Fig. 5.**
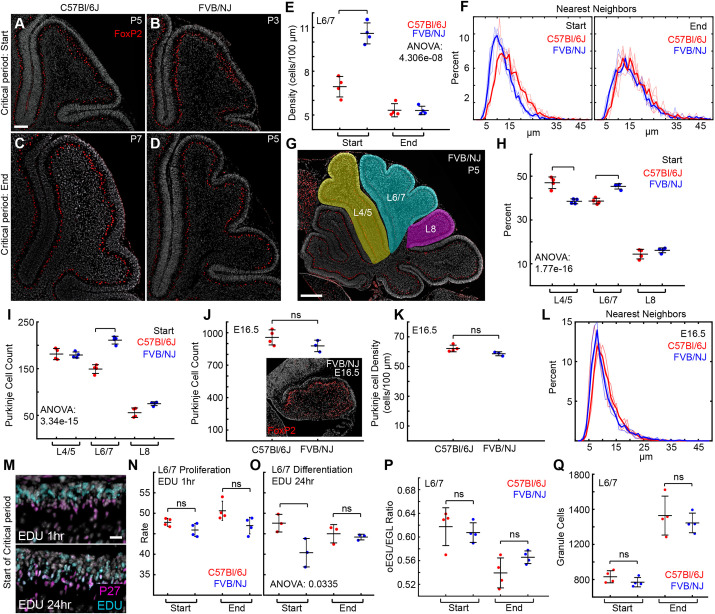
**Purkinje cell number predicts folding amount.** (A-D) Sagittal midline sections of L6/7 at the start and end of the critical period stained with FoxP2 antibody to mark Purkinje cells. Scale bar: 100 µm. (E) Purkinje cell density at the start and end of the critical period in both strains. (F) Nearest neighbor analysis reveals that Purkinje cells are more closely packed in FVB/NJ than in C57Bl/6J at the start of the critical period. Faint lines represent individual samples. (G,H) The percentage of Purkinje cells within L4/5, L6/7 and L8. Pseudocolored image in G highlights the measured regions. Scale bar: 300 µm. (I) L6/7 has fewer Purkinje cells in C57Bl/6J than in FVB/NJ. (J-L) Both strains have similar numbers, densities and distributions of Purkinje cells at E16.5. C57Bl/6J: *n*=4; FVB/NJ: *n*=3. Inset in J shows an example E16.5 FVB/NJ cerebellum stained with FoxP2 antibody to mark Purkinje cells. (M) EGL stained with a marker for differentiating granule cells (P27), the proliferation marker EdU and the nuclear marker DAPI. Scale bar: 20 µm. (N) The proliferation rate is not different between strains. (O) The differentiation rate is lower in FVB/NJ than in C57Bl/6J at the start of the critical period. (P,Q) The relative size of the proliferating layer of the EGL is unchanged between the strains and the total number of cells in the EGL is similar. ns, not significant.

We next measured the density of Purkinje cells in the L6/7 region during the critical period (Fig. 5A-D). At the start of the critical period, the density of Purkinje cells was lower in L6/7 of C57Bl/6J mice compared with FVB/NJ. However, by the end of the critical period, after the folding amount had diverged, the density of Purkinje cells equalized between the strains (Fig. 5E). A similar pattern was observed in L4/5 and L8 (Fig. S8F,G). In addition, the Purkinje cells in L6/7 were tightly distributed with less distance between nearest neighbors in FVB/NJ than in C57Bl/6J at the start of the critical period, before reaching the same spatial distribution at the end (Fig. 5F). Calculating the density of Purkinje cells at both the start and end of the critical period as a ratio of each lobule's final density at P28 showed that L6/7 in FVB/NJ is denser throughout the critical period whereas L4/5 and L8 are only higher at the start in FVB/NJ (Fig. S8H,I).

### There are fewer Purkinje cells in C57Bl/6J L6/7 than in FVB/NJ L6/7 during the critical period

We next addressed whether the difference in Purkinje cell density in L6/7 between strains during the critical period was a result of improper cell sorting or differences in Purkinje cell number. One possibility is that a portion of the Purkinje cells in C57Bl/6J are improperly directed to the lobules surrounding L6/7. To test this hypothesis, we determined the percentage of all Purkinje cells in L4/5, L6/7 and L8 in each strain at the start of the critical period (Fig. 5G,H). In C57Bl/6J, a greater percentage of the Purkinje cells were located within L4/5 and a smaller percentage in L6/7 compared with FVB/NJ (Fig. 5H). Likewise, there were fewer Purkinje cells in L6/7 in C57Bl/6J than in FVB/NJ. However, the number of Purkinje cells was the same between the strains in both L4/5 and L8 (Fig. 5I). We next tested whether fewer Purkinje cells are present at the start of cerebellum folding in C57Bl/6J, specifically those that will occupy L6/7. We found no difference in the number, density or distribution of Purkinje cells between strains (Fig. 5J-L) at E16.5, which is 3 days after Purkinje cells have been born.

### The proliferation rate of granule cells does not correlate with the level of EGL/core differential expansion

Given that the number and density of Purkinje cells are lower in L6/7 in C57Bl/6J compared with FVB/NJ, we hypothesized that the proliferation rate of GCPs might be lower in C57Bl/6J, potentially reducing the level of EGL/core differential expansion. We measured the proliferation rate by injecting 5-ethynyl-2deoxyruidine (EdU) 1 h prior to collection and measured the ratio of EdU^+^ and P27 (Cdkn1b)^−^ GCPs/total P27^−^ GCPs in the exposed surface of L6/7 at the start and end of the critical period (Fig. 5M). Surprisingly, we found no differences between strains in the rate of proliferation at the start or end of the L6/7 critical period (Fig. 5N), suggesting that proliferation within the EGL is not adjusted to set the EGL/core growth ratios or the resulting level of differential expansion in L6/7 between the strains. We then wondered whether FVB/NJ might have a slower rate of differentiation, keeping more GCPs in the proliferative oEGL. We injected EdU 24 h prior to collection and measured the number of GCPs that underwent their final division and migrated to the iEGL within that time period (EdU^+^ and P27^+^ GCPs/total EdU^+^ GCPs) (Fig. 5O). We found that FVB/NJ had a slightly lower rate of differentiation, but only at the start of the critical period. We note that the oEGL/EGL ratio was nevertheless the same between the strains at both time points (Fig. 5P) and the total number of cells in the exposed EGL was the same between the strains. This suggests that the physical arrangement of the cells may play an important role in creating the longer, thinner L6/7 EGL in the FVB/NJ strain and the shorter, thicker L6/7 EGL in the C57Bl6/J strain.

### Cell division angle can predict EGL tangential expansion and thickness

Cell division angle (CDA) within the EGL corresponds to the bias in EGL expansion in the anterior-posterior direction as opposed to the medial-lateral direction during cerebellum development (Legué et al., 2015). Additionally, it was found that removing CHD7 from GCPs affects their division angle, increasing the proportion of cells that divide medial-laterally (Reddy et al., 2021). Under this change, the cerebellum shows lobule-like folds in the medial-lateral axis. Given these previous findings and that the number of cells in the EGL of L6/7 is the same in both strains at the critical period, we hypothesized that CDA could be a fundamental mechanism to regulate both the level of EGL/core differential expansion and the thickness of the EGL, ultimately controlling both the folding amount and folding wavelength.

**Fig. 6.**
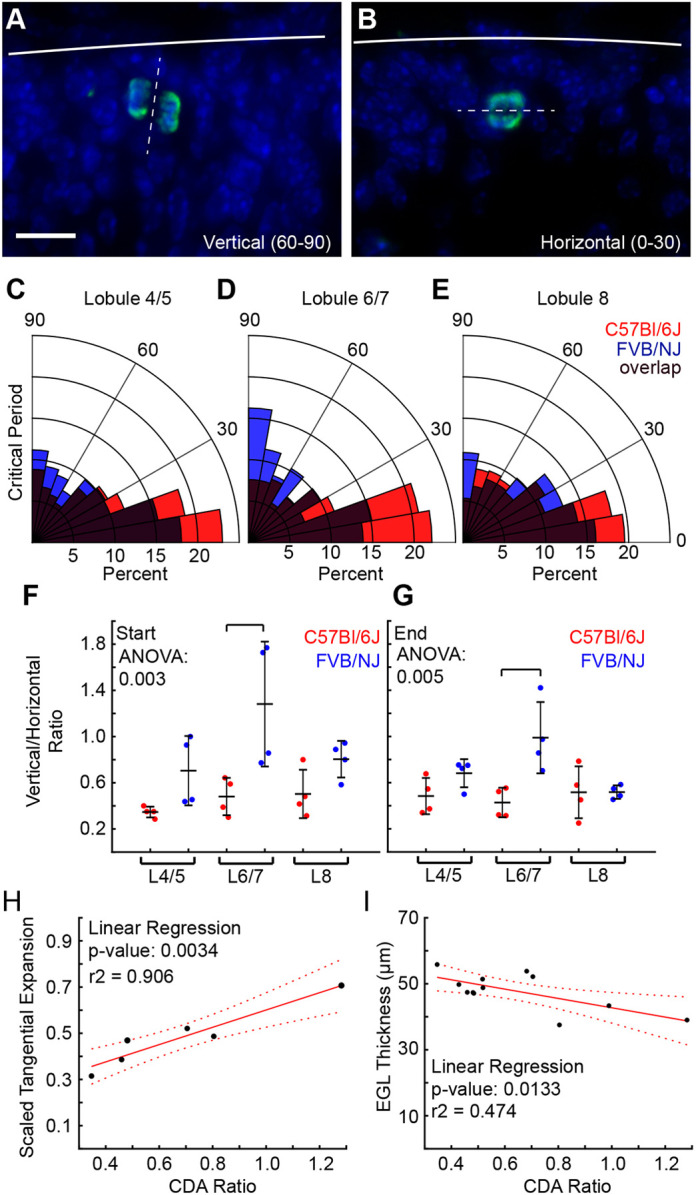
**CDA is altered between strains.** (A,B) PH3 staining showing vertical (A) and horizontal (B) divisions within the EGL. Scale bar: 10 µm. (C-E) Rose plots of CDA in L4/5, L6/7 and L8. *n*=8 per strain. (F,G) Cell division ratio is biased vertically in FVB/NJ compared with C57Bl/6J in L6/7. *n*=4 per strain. (H,I) Cell division ratio predicts tangential expansion and EGL thickness. Solid line: linear regression fit. Dotted line: 95% confidence interval.

We measured the CDA in L4/5, L6/7 and L8 by staining for phospho-histone H3 (PH3) and determining the division plane relative to the surface of the cerebellum (Fig. 6A,B). We predicted that there would be a bias towards vertical divisions (60-90° from the surface) in FVB/NJ given the increased tangential expansion and thinner EGL. Excitingly, we found that, throughout the critical period, FVB/NJ had a higher proportion of vertical divisions in L6/7 compared with C57Bl/6J (Fig. 6C-G, Fig. S9A-C). At the start of the critical period, collectively, FVB/NJ was biased towards vertical divisions (Fig. S9E) compared with C57Bl/6J, but by the end of the critical period the bias towards vertical divisions in FVB/NJ was only maintained in L6/7 (Fig. 6G, Fig. S9A-C).

We next investigated the relationship of CDA and the tangential expansion of the EGL. We measured the average tangential expansion of the EGL in L4/5, L6/7 and L8 in the same cerebella used for the cell division measurements during the critical period. We then ran linear regression analysis using the cell division ratio for each lobule region to predict the tangential expansion of the EGL during the critical period (Fig. 6H). We found a significant fit with a high r^2^ value, demonstrating that the CDA explains over 90% of the variation in the EGL tangential expansion. Similarly, we tested the relationship of the CDA and the EGL thickness. Again, we found a statistically significant relationship between the division angle and the EGL thickness. However, the CDA only explains 47% of the variability. This indicates that the CDA is only one factor, of potentially several, that contribute to the regulation of EGL thickness (Fig. 6I).

## DISCUSSION

**Fig. 7.**
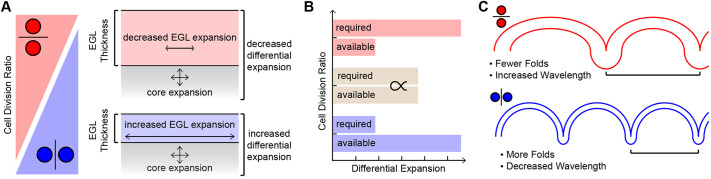
**CDA regulates the level of folding by modulating the tangential expansion of the EGL and its thickness.** (A) High vertical cell division ratios (blue) increase the tangential expansion and decrease the thickness of the EGL. Low vertical ratios (red) increase the thickness of the EGL and decrease the tangential expansion. (B) Increasing the thickness of the EGL and decreasing its tangential expansion increases the force required to fold the tissue and decreases the force available in the tissue. (C) CDA regulates folding amount and wavelength.

Here, we tested multiple predictions for neural tissue folding during cerebellar foliation. We found that the degree of cerebellar folding correlates with regional levels of differential expansion as a result of changes both in the underlying growth ratio between the EGL and the core and in the geometry of the lobules. We also provided developmental evidence that the folding wavelength follows the thickness of the EGL at the time of fissure formation. Although the developing cerebella did not follow a single scaling relationship, as observed across adult cerebral cortices (Mota and Herculano-Houzel, 2015), we suggest that the dynamic regulation of EGL/core differential expansion and EGL thickness during development give rise to the geometry observed in the adult. Lastly, we propose that the angle of cell division within the EGL is a tunable regulator that affects both the tangential expansion and the thickness of the EGL (Fig. 7).

The gene-agnostic approach we took allowed an unbiased analysis of the tissue mechanics predicted to regulate the degree of cerebellar folding. We propose that our approach of comparing inbred mouse strains provides an approximation of the natural folding variation seen between individuals within a species. Intriguingly, the folding differences studied here are similar to those seen across healthy human populations where global variation is minimal and larger changes in the degree of folding are constrained to specific regions (Luders et al., 2006, 2004; Zilles et al., 1988). An alternative approach to ours to capture the mechanical regulation of natural folding variation would be to use an outbred strain that has substantially more variation between individuals than inbred strains. However, without the folding robustness seen in C57Bl/6J and FVB/NJ, a direct comparison of different individuals at different developmental time points would be impossible.

The time-agnostic analysis of cerebellar growth rate (Fig. 2A) allowed us to identify a critical period during development, intrinsic to each strain, when the tissue mechanics diverged. Given that the clock-based rate of development runs slower in C57Bl/6J than in FVB/NJ mice, a dependence of clock-time would have reduced the power of the comparisons between the strains. The fact that FVBN/J reaches the same size and degree of folding ∼2 days prior to C57Bl/6J illustrates that the folding amount is intrinsic to the tissue mechanics of the cerebellum and not the global speed of development.

We uncovered differences in the lobule EGL/core growth ratios and levels of differential expansion between FVB/NJ and C57Bl/6J. First, the tangential expansion of the EGL was greater in FVB/NJ compared with C57Bl/6J in all lobules measured. The differences in L6/7 between the strains was further magnified by the slight geometric difference in the starting shape of L6/7 in the two strains. This shape difference determines the required ratio of EGL to core growth needed to achieve the same differential expansion that leads to folding. In both strains, L4/5 and L8 showed very similar EGL/core growth ratios to L6/7. Yet the divergent geometries significantly modulate the resulting level of differential expansion needed to produce additional folding. Our data indicate that control of both the EGL/core growth ratios and the lobule geometries are regionally dictated to produce the correct level of cerebellar folding.

The thickness of the EGL during the critical period also varied within and between the strains, as well as within individual lobules. Excitingly, the significantly greater thickness of the L6/7 EGL in C57Bl/6J compared with FVB/NJ mice, at the time when the fissures surrounding L7 form, correlates with a longer wavelength of the resulting lobule. In contrast, L8, which showed no difference in the wavelength between strains at P28, had a similar EGL thickness at the time of fissure formation. The slight difference in thickness detected is a fraction of a single-cell diameter and is likely not biologically significant.

### Purkinje cell density during the critical period predicts folding amount

The smaller number and density of Purkinje cells in L6/7 of C57Bl/6J at the start of the critical period correlates with the future degree of folding (Fig. 5E,I). Given that the cerebella of the two strains have the same number of total Purkinje cells in the midline at E16.5 and that the lobules adjacent to L6/7 have comparable numbers of Purkinje cells as their FVB/NJ counterparts during the critical period, we hypothesize that the Purkinje cells in the L6/7 region of C57Bl/6J undergo increased cell death or move a greater distance medial-laterally compared with FVB/NJ mice later in development.

Surprisingly, Purkinje cell densities at P28 were elevated in C57Bl/6J compared with FVB/NJ. This shows that FVB/NJ achieves more growth per Purkinje cell than C57Bl/6J and suggests that there may be strain-intrinsic differences in the degree to which Purkinje cells are able to drive the expansion of other cell populations to scale the proper balance of cells within the cerebellum (Joyner and Bayin, 2022; Willett et al., 2019). Indeed, there are many fixed mutational differences between C57Bl/6J and FVB/NJ mice, and, indeed, variations also between sub-strains (Bowes et al., 1990; Kasugai et al., 2007; Mekada and Yoshiki, 2021; Schauwecker, 2012; Timmermans et al., 2017). Together, our findings suggest that both the regional number of Purkinje cells during development, as well as their strain intrinsic effectiveness, could have roles in scaling the folding and the circuitry.

Given the differences in the Purkinje cell numbers as well as the role of GCPs in the growth, expansion and folding of the cerebellum, we tested whether the level of GCP proliferation is a tunable regulator of the EGL/core growth ratio and therefore of differential expansion of layers and folding (Corrales et al., 2006; Legué et al., 2016). Surprisingly, our data indicate that the proliferation rate within the EGL of L6/7 is not tuned to adjust the level of tangential expansion of the EGL (Fig. 5N). Indeed, the similarity in the number of cells within the EGL between the strains before and after the folding differences arise, strongly suggests that the rates of proliferation or differentiation are less important than the arrangement of cells within the EGL.

### CDA as a tunable regulator of the tangential expansion and thickness of the EGL

In *Lkb1* (*Stk11*) mutant mice, cerebellar folding is increased and EGL thickness is decreased without a change in GCP proliferation rate, but with an increase in vertical cell divisions (Ryan et al., 2017). Additionally, lobule-like structures form in the mutant cerebellum in the medial-lateral direction when the CDA is biased in that direction (Reddy et al., 2021). By shifting the CDA, the cerebellum may adjust various mechanical parameters that regulate the degree and wavelength of folding. A horizontal bias should increase the thickness of the EGL at the expense of its tangential expansion, leading to an increase in the force required to fold. Conversely, a reduction in tangential expansion will reduce the level of EGL/core differential expansion, the driving force for folding, and reduce folding (Fig. 7).

Supporting this model, the angle of cell division in the EGL is preferentially biased vertically in L6/7 of FVB/NJ compared with C57Bl/6J precisely when we measured a thinner EGL and greater tangential-expansion of the EGL in FVB/NJ (Fig. 6C-G). Further, at the start of the critical period in FVB/NJ, there is an increased bias to vertical divisions compared with C57Bl/6J and each lobule region in FVB/NJ has a higher tangential expansion than in C57Bl/6J (Fig. 3A, Figs S5A,B, S9E). Similarly, at the end of the critical period, when L4/5 and L8 have similar CDAs within and between the strains, the resulting EGL thicknesses within and between the strains are similar (Figs S7A, S9A,C).

Although we detected a statistically significant relationship between the angle of cell division and the EGL thickness, it only accounts for 47% of the observed variation in EGL thickness. This result predicts that other factors work together with the division angel to tune the EGL thickness. It is plausible that the migration mechanics of GCPs, or their rates of differentiation, could play a role, or it may be that tensile forces, predicted to cross the EGL, also affect the EGL thickness (Lawton et al., 2019). Strikingly, the CDA predicts over 90% of the variation in the tangential expansion of the EGL, suggesting that it is a dominant regulator of the tangential expansion and, by extension, folding amount of the cerebellum.

## MATERIALS AND METHODS

### Animals

Animals were maintained in accordance with protocols approved by the Institutional Animal Care and Use Committees at Mississippi State University and Memorial Sloan Kettering Cancer Center. All data were collected from the two inbred mouse strains C57Bl/6J (The Jackson Laboratory, 000664) and FVB/NJ (The Jackson Laboratory, 001800). Both sexes were used for analysis. Mice were maintained on a 12 h light/dark cycle and food and water were provided *ad libitum*.

The appearance of a vaginal plug was used to mark noon as E0.5. Pups were injected subcutaneously with 25 μg/g EdU (A10044, Invitrogen) one, or 24 h prior to collection for proliferation and differentiation measurements, respectively.

### Tissue preparation and imaging

#### Adult stage

P28 cerebella were dissected out of the skull at noon and fixed in Bouin's solution at room temperature and rotated for 24 h. Brains were then washed in PBS, dehydrated, and prepared for paraffin embedding. Parasagittal sections were collected with a Leica Microtome RM2235 at 8 µm thickness.

#### Developmental series

All cerebella of developmental stages were collected at approximately noon on the day of collection. Post-natal brains were dissected out of the skull and postfixed in fresh, ice-cold 4% paraformaldehyde for 24 h. Embryonic heads were postfixed as above. Brains were washed in PBS and cryoprotected with washes of 15% sucrose and 30% sucrose in PBS. Brains were frozen in OCT (23-730-571, Thermo Fisher Scientific), stored at −80°C and cut with a Leica CryoStat (CM3050s) to obtain 10-µm-thick parasagittal sections.

The slides with the most midline sections were chosen and stained with Hematoxylin & Eosin (H&E). All sections were imaged on a Zeiss Observer Z.1 with Apatome, or Leica Thunder Imaging System.

### Area, length, positive curvature, folding index measurements, and cortical thickness

Measurements were collected either in Imaris (Bitplane) or ImageJ as previously described (Lawton et al., 2019). Three midline sections were measured from each cerebellum and the median values were reported. The global pial surface length was measured from the anterior start of the surface of the molecular layer (P28) or the anterior start of the EGL (developmental series). The cross-sectional area (area of the midline sagittal section of the cerebellum) was measured by joining the anterior and posterior ends by following the ventricular zone. In ImageJ, the positive curvature (the convex hull, a measure of the exposed surface) was created using the convex hull tool in ImageJ to delete all negative curvature points. Regional, lobule-based measurements were similarly collected. Lobule lengths and their positive curvature lines were measured from the surface of the molecular layer/EGL at the AC to the anterior of the lobule to the center of the AC posterior to the lobule region. Lobule region areas were measured starting at the base of the EGL directly below the surface of the AC anterior to the lobule region of interest. The area then followed the surface of the EGL until it reached the posterior AC enclosing the lobule region. The area was extended directly below the surface of the AC to the bottom of the EGL. Then, the area was directly closed by connecting to the start. The folding index was calculated as [1−(positive curvature/surface length)]×100 (Lawton et al., 2019).

To test the for the presence of a universal scaling relationship as reported in the cerebral cortex, we estimated the ‘cortical thickness’ by dividing the cerebellar area by the pial surface length. The square root of the thickness multiplied by the pial surface length was then used to predict the positive curvature.

For the adult stage, five brains were measured per strain. For the developmental series, C57Bl/6J were collected daily from E16.5 to P9 and FVB/NJ were collected daily from E16.5 to P7. For the developmental series, 94 cerebella were measured for C57Bl/6J and 96 for FVB/NJ. For each brain in the series, the two or three most midline sections were measured, and the median values were reported.

### Antibodies and EdU staining

Antibody and EdU staining was performed as previously described (Lawton et al., 2019). Primary antibodies were incubated overnight at 4°C or at room temperature for 4 h. Primary antibodies used were: goat anti-Foxp2 (1:1000; EB05226, Everest Biotech), rabbit anti-PH3 (1:1000; 06-570, EMD Millipore), mouse anti-P27 (1:500; 610241, BD Biosciences), rabbit anti-calbindin (1:500; CB38, Swant). All antibodies were diluted in 2% milk and 0.2% Triton X-100. Alexa Fluor-conjugated secondary antibodies (A32795TR, A21432, A31570, A21206, Invitrogen) were used at 1:1000 and sections were counterstained with DAPI.

### Cell counts, proliferation, differentiation, EGL thickness, division angle

Purkinje cells were counted in Imaris. Four cerebella per strain and per time point for developmental stages were stained with FoxP2 antibody to mark Purkinje cells. For each brain, six to nine midline sections were measured. At P28, five brains were stained with calbindin antibody to mark Purkinje cells and four midline sections were measured per brain. The median value for each brain was reported. For E16.5 measurements, four brains were used for C57Bl/6J and three for FVB/NJ and three sections per brain were measured. The nearest neighbor analysis was calculated in Imaris software. MATLAB (MathWorks) software was used to remove the duplication bias that arose when a pair of cells is nearest neighbors with themselves.

The proliferation rate was measured as previously described (Lawton et al., 2019). Four cerebella were used per each strain and per time point. Three midline sections per brain were measured. The median proliferation rate for each brain was reported. All cells within the EGL of the exposed portion of the lobule were measured. DAPI^+^ cells that were both EdU^+^ and P27^−^ were counted as proliferating. The proliferation rate was calculated as (number of EdU^+^ cells)/(number of DAPI^+^; P27^−^ cells). Similarly, the differentiation rate was calculated as (number of EdU^+^; P27^+^ cells)/(number of P27^+^ cells). Three midline sections per brain were measured and three brains per age and genotype were measured.

The thickness of the EGL was first measured by dividing the area of the EGL by its length. In the L6/7 region, the thickness was also measured in the exposed and covered portions of the EGL. Lastly, the thickness and density of the exposed region of L6/7 was measured by counting all the cells within the EGL and dividing the counts by the length of the exposed surface.

The CDA was measured in Imaris software. Four brains were measured per strain and per time point. For each brain, six to nine midline sections were measured. The median values per brain were reported. The angle of cell division was measured in relation to the local surface of the EGL.

To test the relationship between CDA and EGL tangential expansion, the CDA was measured in L4/5, L6/7 and L8 in four brains at the start of the critical period and the average of the four brains was taken for L4/5, L6/7 and L8. The EGL length was also measured in those brains as well as the four brains at the end of the critical period. The average length difference between the start and end for each lobule region was normalized to control for the differences in their starting sizes. The six average cell division ratios (L4/5, L6/7, L8 from C57Bl/6J and FVB/NJ) from the start of the critical period were plotted against the normalized EGL expansion during the critical period. Similarly, the average cell division ration for each lobule region at the start and end of the critical period was plotted against the average EGL thickness measured in the same brains at the start and end.

### Size-free wavelength measurements

We used MorphoJ software to run a standard landmark-based procrustean analysis to control for the size difference between the strains (Klingenberg, 2011). We used the nine conserved ACs as the landmarks and ran standard alignments individually and with strains together.

### Statistics

All statistics analyses were run using MATLAB (MathWorks). When individual comparisons were made, the two-sample *t*-test was used. The statistical threshold was a *P*<0.05. When multiple comparisons were being made, one-way ANOVA analyses were performed. After ANOVA analysis, a multiple comparison was run with Tukey's honestly significant difference criterion. The statistical threshold was set at *P*<0.05. When testing for differences between the strains, the lobule regions, and any interactions, two-way ANOVA analyses was performed and the *P*-values for strain, lobule and interaction were reported. All error bars represent s.d. No statistical methods were used to predetermine the sample sizes. We used sample sizes aligned with the standards in the field. No randomization was used nor were data collected or analyzed by operators unaware of the groupings/treatment. See Table S1 for statistical data.

### Regression analyses

For the global growth curve fitting, we ran non-linear regressions using a basic form of the Gompertz function:
(1)

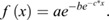
Initial coefficients for the equation were estimated through repeated graphical exploration and Excel solver to minimize residuals. Analysis was carried out in MATLAB.

To test for a universal scaling relationship between the exposed surface and the thickness and total surface, we ran nonlinear regressions for a power equation as previously reported (Mota and Herculano-Houzel, 2015):
(2)

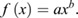
Linear regression analysis was performed in MATLAB. Multiple linear regression analysis was carried out to test for differences between slopes. See Table S1 for statistical data.

## Supplementary Material



10.1242/develop.202184_sup1 Supplementary information

## The people behind the papers

This article has an associated ‘The people behind the papers’ interview with some of the authors.
