# Supplementary material for: Cell division angle predicts the level of tissue mechanics that tune the amount of cerebellar folding
Source: Development. 2024 Feb 13;151(3):dev202184. doi: 10.1242/dev.202184 (PMC10911135; doi:10.1242/dev.202184)
Supplement: Supplementary information [file develop-151-202184-s1.pdf]

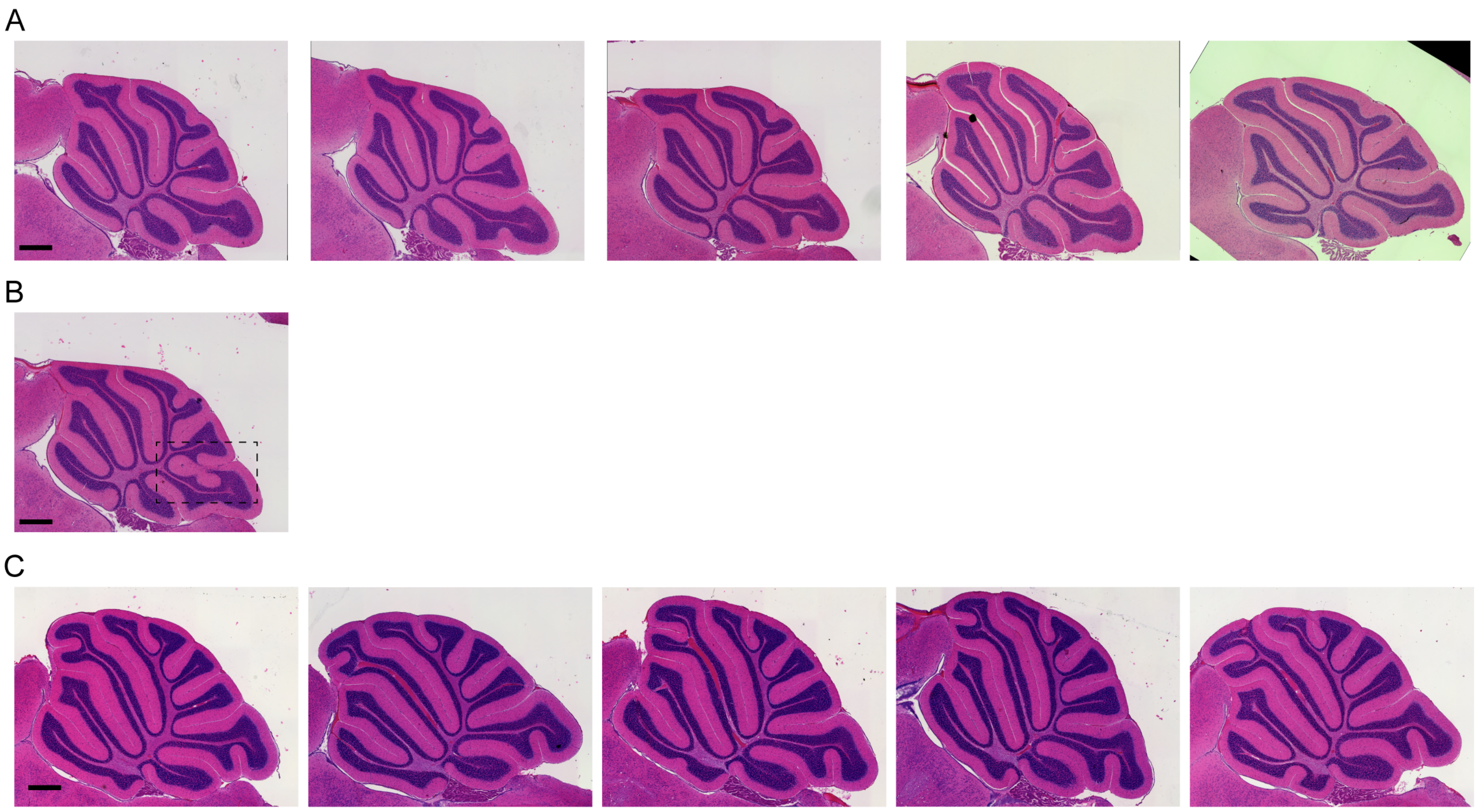

Supplemental Figure 1

Fig. S1. C57Bl/6J and FVB/NJ cerebella at P28 have robustly different levels of folding at the midline of the vermis. A) Sagittal midline sections of 5 C57Bl/6J cerebella **B)** C57Bl6/J cerebella showing heterotopia between lobule 8 and Lobule 9. **C)** Sagittal midline sections of 5 FVB/NJ. All cerebella were stained with H&E. One example from A and C is shown in Fig. 1A and B, respectively. Scale Bars: 0.5 mm.

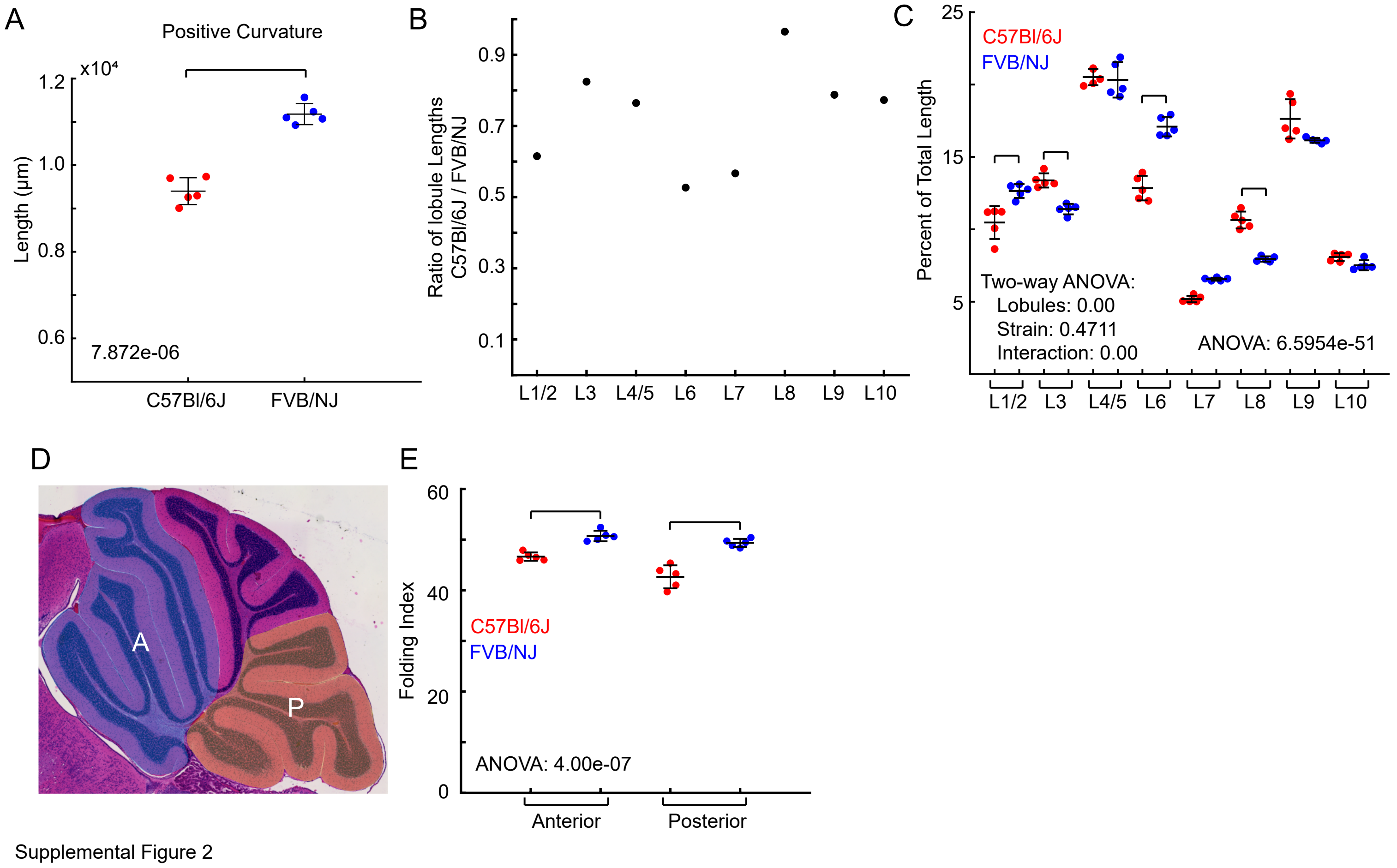

Fig. S2. Size difference between C57Bl/6J and FVB/NJ is regionally regulated. A) Positive curvature. P-value reported. **B)** Ratio of C57Bl/6J lobule lengths over FVB/NJ lobule lengths. **C)** Lobule lengths as a percentage of total length. Brackets indicate statistical differences. **D)** Image showing anterior (blue shading) and posterior (yellow shading) regions of cerebellum. **E)** Folding index of anterior and posterior regions. Brackets indicate statistical differences.

Development • Supplementary information

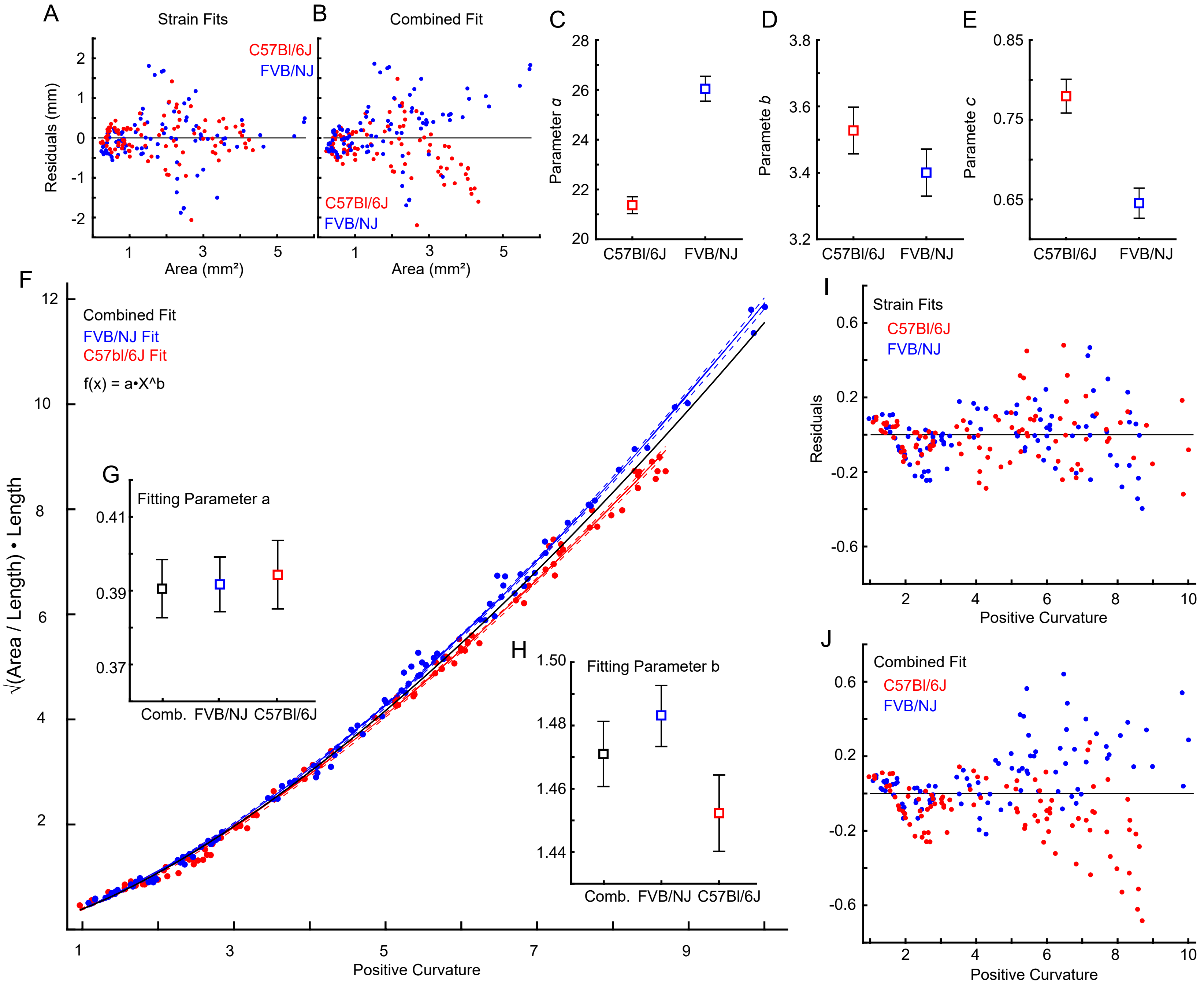

Fig. S3. Global ratio of growth. A) Residuals from fitting each strain to individual Gompertz function (see Fig. 2A). **B)** Residuals are poorly patterned and larger when data is combined and fitted. **C-E)** The three parameters of the individual Gompertz function fittings are distinct between the strains. **F-I)** Strain have different relationships between the positive curvature and the thickness and pial surface length. **J-K)** Residuals are poorly patterned and larger when data is combined and fitted.

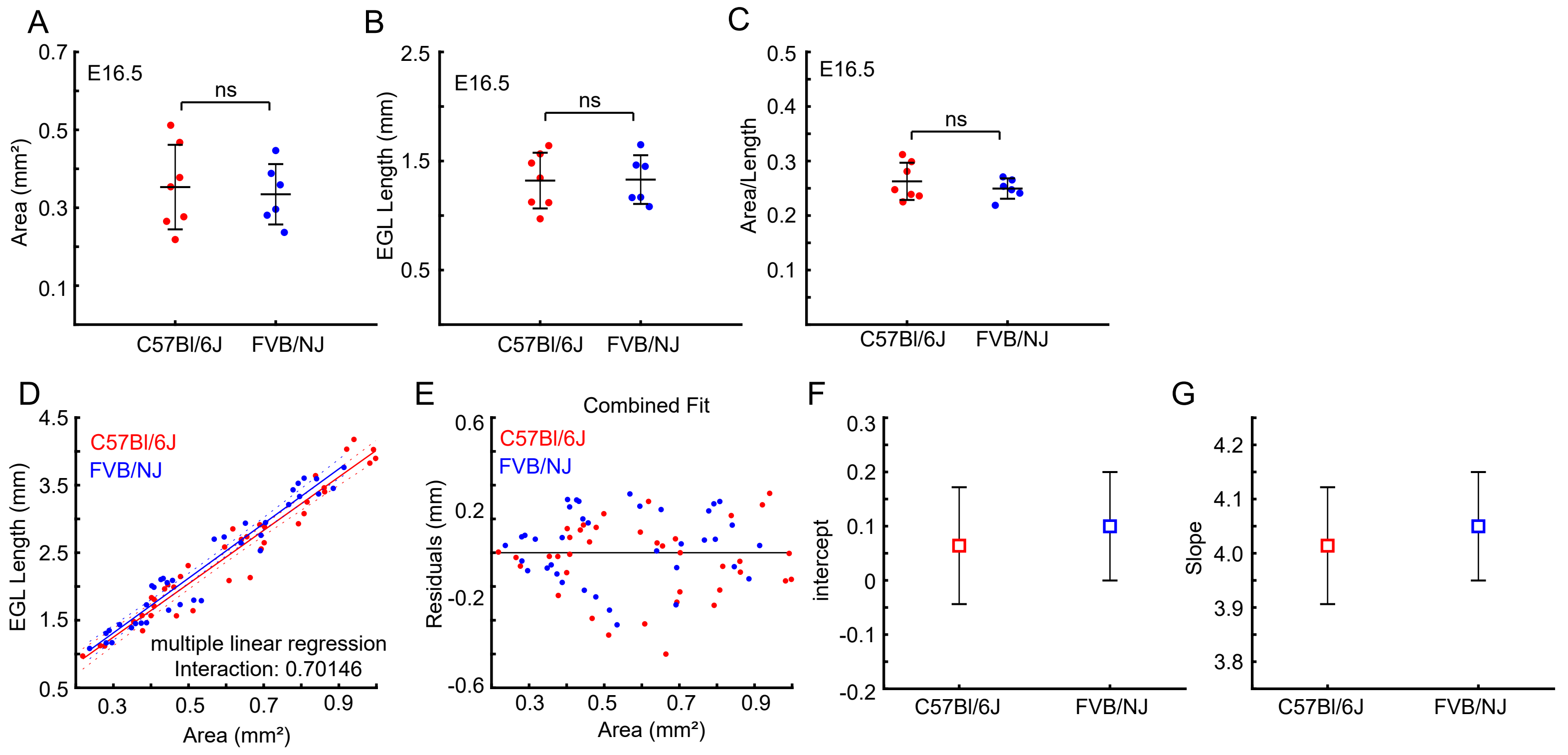

Fig. S4. Global ratio of growth is similar during initiation of folding. A-C) At E16.5 the area (p-value 0.7353), length (p-value: 0.9473), and the ratio of the area and length (p-value 0.417) are unchanged between the strains. **D)** Growth ratio from the start (E16.5) to 1mm2 (~P0). Multiple linear regression analysis shows no difference between the slopes. A subset of the FVB/NJ data was previously published (Lawton et al., 2019) **E)** Residuals are small and well patterned with a single combined fit. **F,G)** The parameters of the individual fittings are overlapping showing no difference between the strains at this early period of growth. For full statistics see statistics table.

Development • Supplementary information

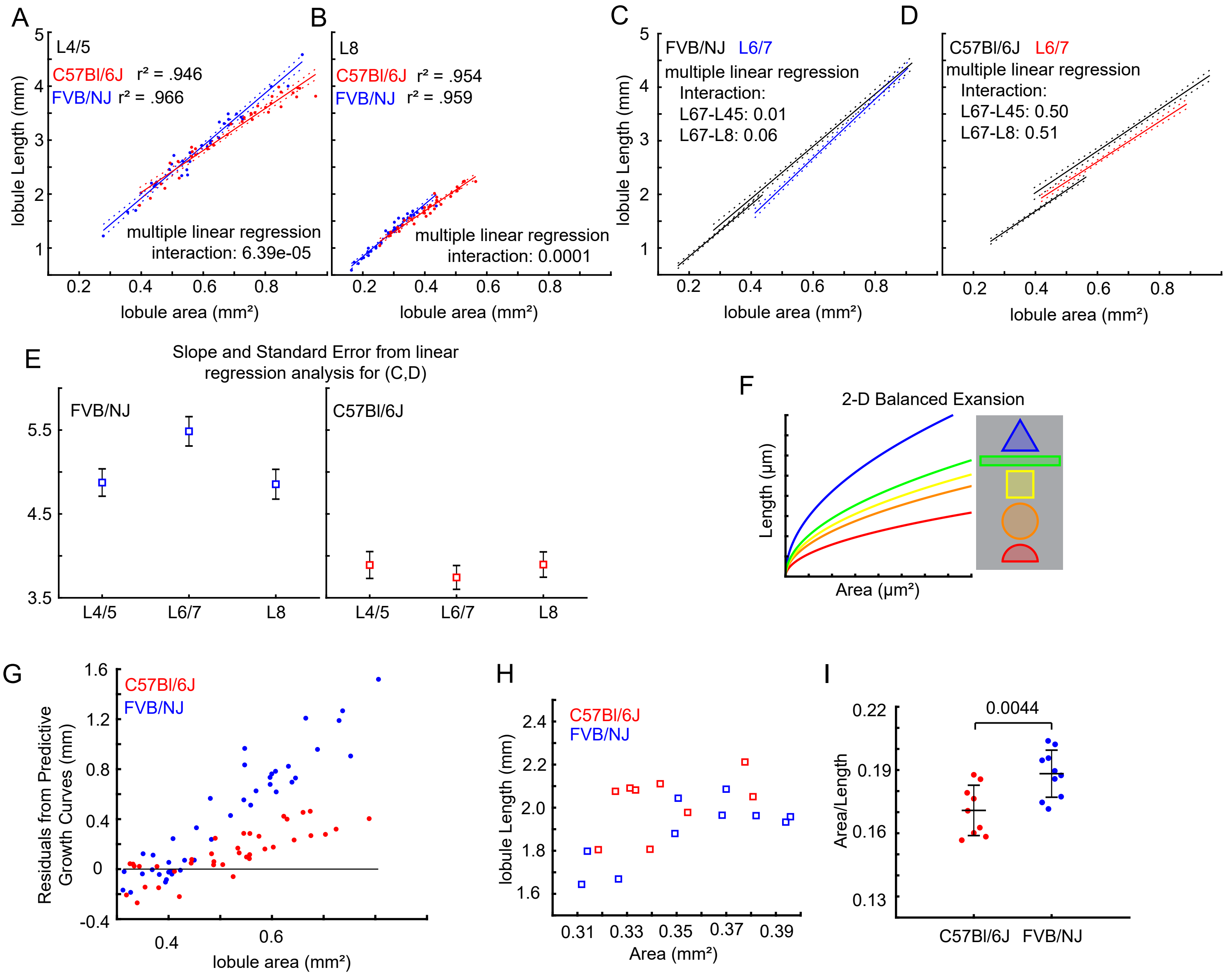

Fig. S5. Lobule growth ratios are higher in FVB/NJ than in C57Bl/6J and the resulting differential-expansion is dependent of the geometry of the lobule. A) Multiple linear regression analysis of L4-5 region shows statistical difference between the strains. R-squared values reported for individual fits. **B)** Multiple linear regression analysis of L8 region shows difference between the strains. R-squared values reported for individual fits. **C-D)** Multiple linear regression analysis within each stain. **E)** Calculated slope parameters from linear regression analysis of L4-5, L6-7, and L8 for both strains **F)** Cartoon depicting balanced growth ratio (length/Area) curves for common 2-D shapes. **G)** The residuals calculated from the predictive growth curves show that the growth ratio of C57Bl/6J is more similar to its balanced growth curve than FVB/NJ. **H,I)** L6-7 has a slight difference in geometry between the strains with C57Bl/6J having more length per area than FVB/NJ.

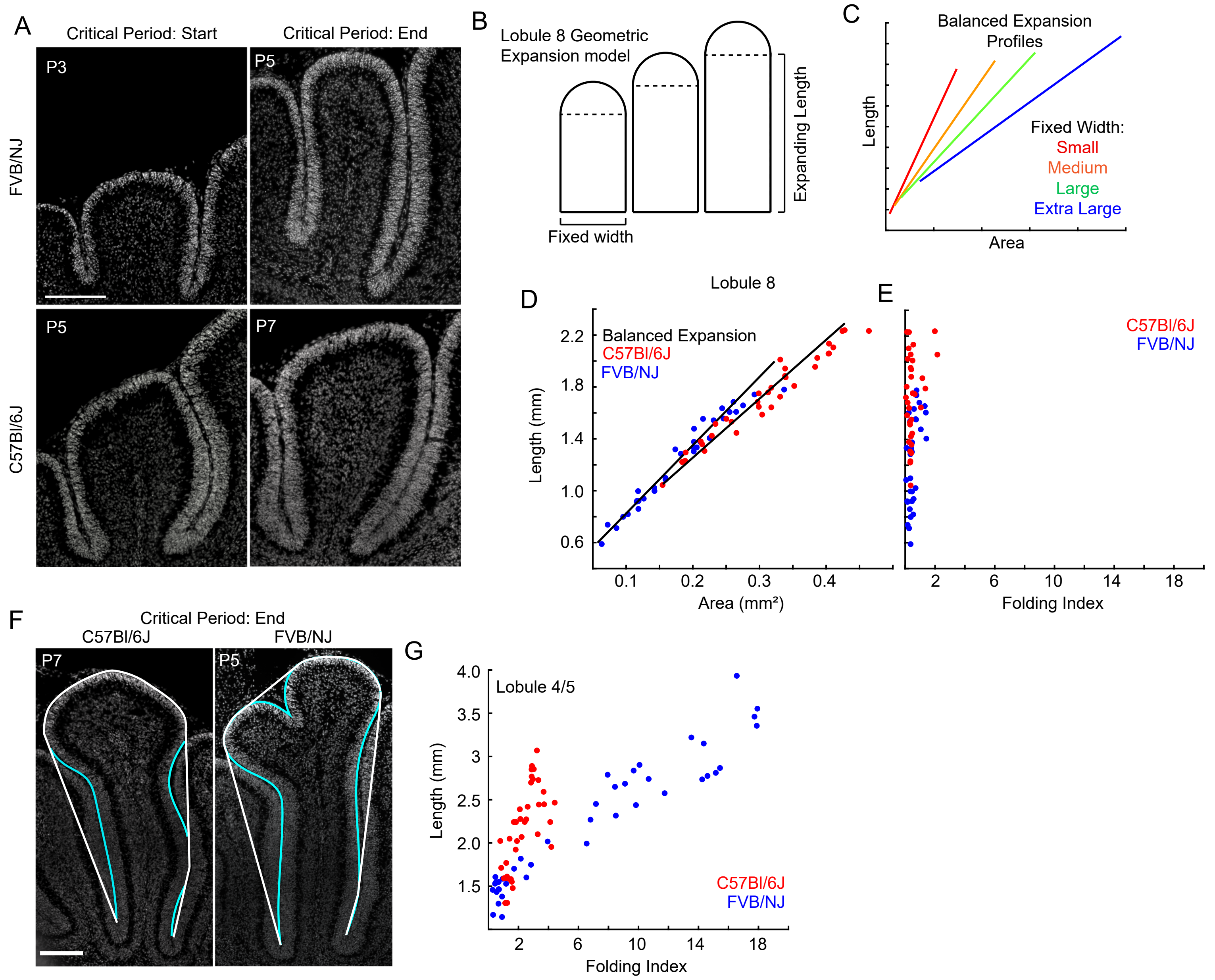

Fig. S6. Lobule geometry regionally regulates folding amount. A) Sagittal midline sections of L8 region stained with Dapi at the start and end of the critical period in FVB/NJ and C57Bl/6J showing the constraints from the surrounding lobule regions and the limited exposed surface. Scale bar: 200µm **B)** Model of constrained expansion. The width of the lobule which sets the parameters for the semi-circle is constrained while the length is allowed to expand. **C)** Balanced expansion curves for such growth are linear and the slope decreases as the fixed width is increased. **D)** The growth ratio of L8 is well predicted in C57Bl/6J and FVB/NJ by this type of constrained growth showing no evidence of differential-expansion. **E)** The folding index shows that L8 in both strains remains unfolded as its growth ratios remain balanced. **F)** Sagittal midline sections of L45 stained with Dapi at the end of the critical period. Cyan line: EGL length. White line: Positive curvature. Scale bar: 200µm **G)** Folding index of L45 through the critical period. While L4-5 in C57Bl/6 remains unfolded the measured increase in folding index comes from the complex shape of the lobule region.

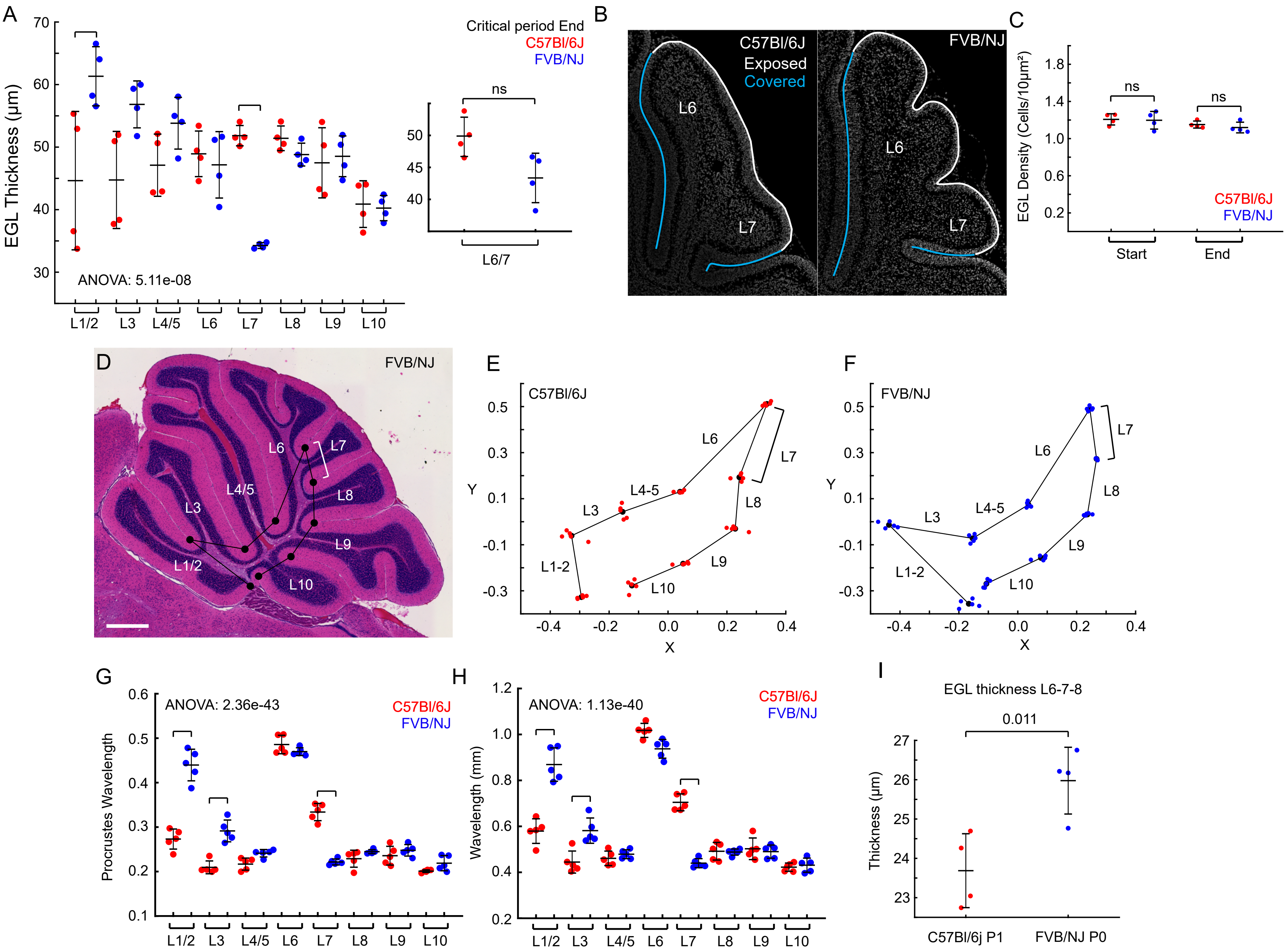

Fig. S7. EGL thickness is regionally varied within the cerebellum and correlates with folding wavelength. A) EGL thickness at end of critical period. Brackets indicate regions with statistical difference. See Statistics Table for full analysis. **B)** Midline sagittal sections of C57Bl/6J and FVB/NJ L6-7 stained with Dapi. Cyan line: covered EGL. White line: exposed EGL surface. **C)** EGL density in exposed surface. ANOVA p-value: 0.2534. **D)** Midline sagittal section of FVB/NJ at P28 showing landmarks placed at the conserved anchoring centers. Black lines show lobule wavelengths. White bracket indicates L7 wavelength. Scale bar: 0.5mm **E,F)** Individual Procrustes alignments of landmarks of C57Bl/6J and FVB/NJ bracket indicates L7 wavelength. **G)** Wavelengths from Procrustes alignment for each lobule region. Brackets indicate statistical differences. See Statistics table for full analysis. **H)** Wavelengths from real distances. Brackets indicate statistical differences. See statistics table for full analysis **I)** EGL thickness in L6-7-8 region at P0 and P1 for FVB/NJ and C57Bl/6J.

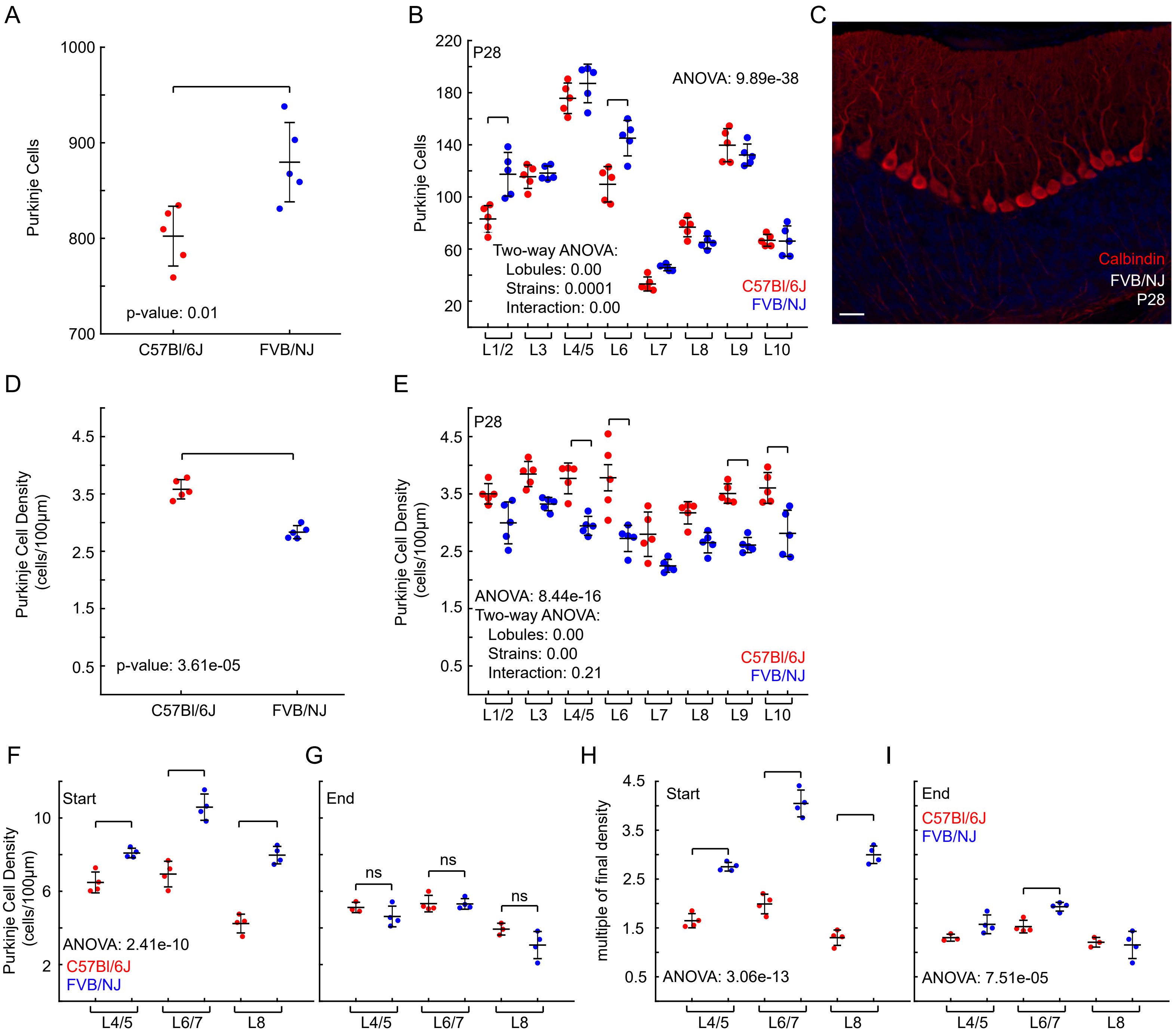

Fig. S8. Purkinje cell density is regionally regulated in the Cerebellum and different between the strains. A,B) Number of Purkinje cells P28. Brackets indicate statistical differences. **C)** Sagittal midline section of FVB/NJ at P28 stained with Calbindin and Dapi to mark the Purkinje cells. Scale bar: 50µm **D,E)** Purkinje cell density at P28. C57Bl/6J has higher density of Purkinje cells even in L6 that has a reduced number compared to FVB/NJ. Brackets indicate statistical differences. **F,G)** Purkinje cell density during the critical period in L4-5, L6-7, and L8. Brackets indicate statistical differences. ns = not statistically significant. **H,I)** Purkinje cell density during the critical period as a multiple of final density at P28. Brackets indicate statistical differences.

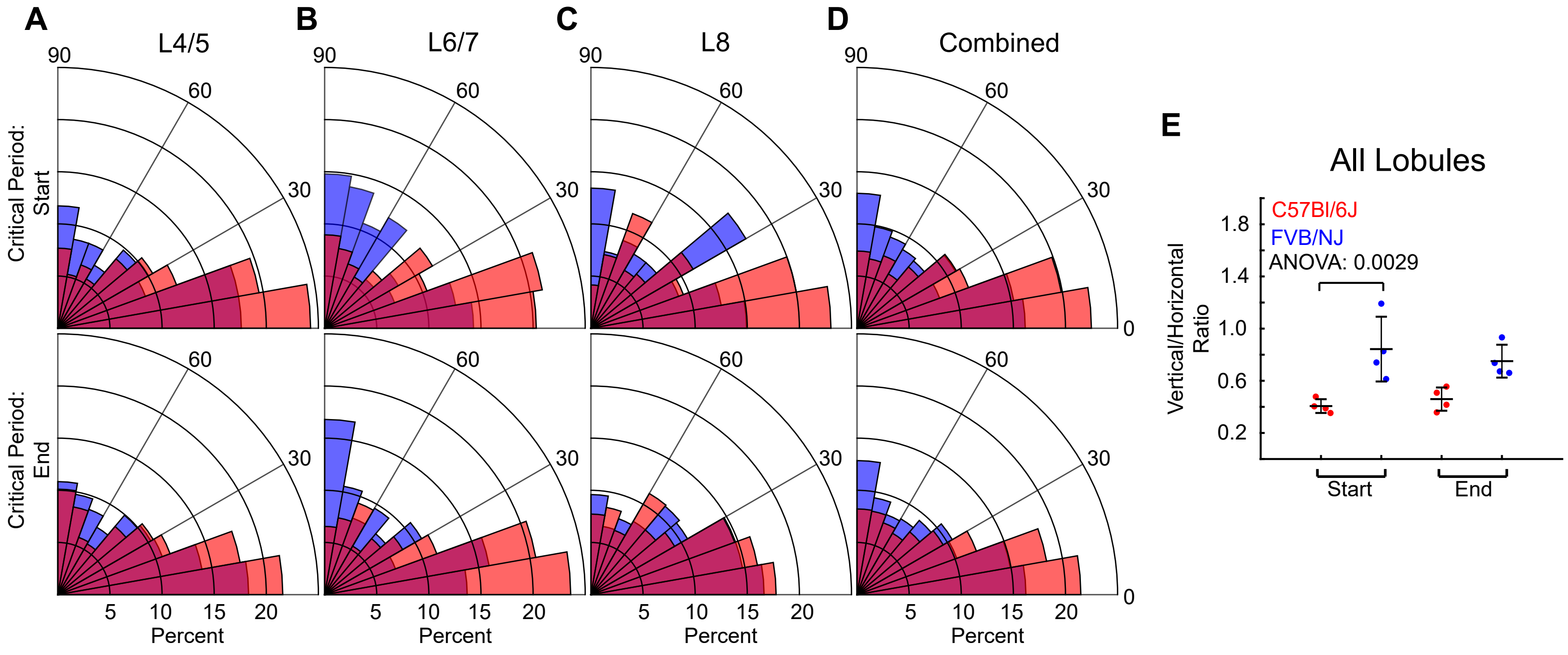

Fig. S9. Cell Division Angle is regionally adjusted during the critical period. A-C) Polar plot of cell division angles measured in L4-5, L6-7, and L8 at the start and end of the critical periods. At the end of the critical period the difference between the strains is mostly contained to L6-7. **D)** Combined cell division angles measured from L4-5, L6-7, and L8. **E)** Cell division angle ratio of combined data. Bracket indicates statistical difference.

Table S1. Statistics table

Available for download at
https://journals.biologists.com/dev/article-lookup/doi/10.1242/dev.202184#supplementary-data

Development • Supplementary information
