# Supplementary material for: Cell division angle predicts the level of tissue mechanics that tune the amount of cerebellar folding
Source: Development. 2024 Feb 13;151(3):dev202184. doi: 10.1242/dev.202184 (PMC10911135; doi:10.1242/dev.202184)
Supplement: Supplementary file 1 [file develop-151-202184_review_history.pdf]

Cell division angle predicts the level of tissue mechanics that tune the amount of cerebellar folding

Amber G Cook, Taylor V Bishop, Hannah R Crowe, Daniel N Stevens, Lauren Reine, Alexandra L Joyner and Andrew K Lawton
DOI: 10.1242/dev.202184

Editor: Debra Silver

Review timeline
Original submission: 17 July 2023
Editorial decision: 18 September 2023
Rebuttal submitted: 3 October 2023
Rebuttal response: 8 October 2023
First revision received: 19 November 2023
Accepted: 10 January 2024

Original submission

First decision letter

MS ID#: DEVELOP/2023/202184

MS TITLE: Cell division angle regulates the tissue mechanics and tunes the amount of cerebellar folding

AUTHORS: Amber G Cook, Taylor V Bishop, Hannah R Crowe, Daniel N Stevens, Lauren Reine, Alexandra L Joyner, and Andrew K Lawton

I have now received all the referees reports on the above manuscript, and have reached a decision. The referees' comments are appended below, or you can access them online: please go to BenchPress and click on the 'Manuscripts with Decisions' queue in the Author Area.

As you will see from their reports, the referees recognise the potential of your work, but they also raise significant concerns about it. Given the nature of these concerns, I am afraid I have little choice other than to reject the paper at this stage.

However, having evaluated the paper, I do recognise the potential importance of this work. I would therefore be prepared to consider as a new submission an extension of this study that contains new experiments, data and discussions and that address fully the major concerns of the referees. The work required goes beyond a standard revision of the paper.Please bear in mind that the referees (who may be different from the present reviewers) will assess the novelty of your work in the context of all previous publications, including those published between now and the time of resubmission.

Reviewer 1 requests additional investigation of cell fate and migration. Reviewer 2 notes a lack of rigor and need for significant additional mathematical analysis. The reviewers also request clarity of language and I would note the title of your study as well as some conclusions currently implies a causal relationship which is not shown. As such, I do agree with reviewer 1 that analysis of some mutant foliation patterns would really bolster conclusions. Apologies for the long review process however difficulties with getting 1 review on time coupled with difficulties finding suitable reviewers delayed this process.

© 2024. Published by The Company of Biologists under the terms of the Creative Commons Attribution License (https://creativecommons.org/licenses/by/4.0/).

Reviewer 1

Advance summary and potential significance to field

In this manuscript, Cook et al, study folding and foliation in the cerebellum and determine the cellular basis of this enigmatic phenomenon. The authors test multiple predictions for neural tissue folding during cerebellar foliation including external granule layer (EGL) thickness, EGL proliferation, Purkinje cell density, and finally, plane of cell division. Based on their rigorous analyses they discover that plane of cell division is the only factor that contributes to the regulation of EGL thickness.

Comments for the author

In this manuscript, Cook et al, study folding and foliation in the cerebellum and determine the cellular basis of this enigmatic phenomenon. The authors test multiple predictions for neural tissue folding during cerebellar foliation including external granule layer (EGL) thickness, EGL proliferation, Purkinje cell density, and finally, plane of cell division. Based on their rigorous analyses they discover that plane of cell division is the only factor that contributes to the regulation of EGL thickness.

This is an exceptionally well-written manuscript on an important aspect of cerebellar development that has bewildered researchers in the field. The authors have carefully considered multiple possibilities and tested each one of them.

While this paper would have benefitted from analysis of mice with foliation defects, I hope this is being pursued as a separate study.
While I strongly recommend this manuscript for publication, the following points need to be clarified:

1. For a significant portion of the manuscript, the EGL is assumed to be a single entity. Later in the manuscript, the EGL is described as having a combination of outer proliferative and inner post-mitotic cells. When the authors describe folding wavelength being regulated by adjusting EGL thickness, does the composition of the EGL (proliferating vs differentiating cells) matter or is it entirely based on thickness irrespective of whether there is greater proliferation vs differentiation?

2. Since plane of cell division seems to be the major determining factor of EGL thickness, does plane of division have any influence on subsequent cell fate (renewal vs differentiation) between the two strains? In the cerebral cortex vertical divisions are often associated with self-renewal whereas horizontal divisions are often asymmetric.

3. Could there be migratory differences between the two strains, with cellular movement being in the direction of oEGL to inner EGL within C57, while FVB exhibiting more tangential movement?

4. The authors surmise that a higher density of Purkinje cells in FVB could result in an increased secretion of mitogen, Sonic hedgehog. Considering that there is no difference in proliferation levels between the two strains how would this be likely? This sentence should either be excluded or an ISH for Shh or any readouts of the Shh pathway need to be included to prove their point.

5. Minor: Line 52: Replace arraigned with arranged; Line 109: Replace Fig 2E with 1E; Line 172: Define SDF and IF; For Figure 4A, and B, include images from the end of the critical period as well.

Reviewer 2

Advance summary and potential significance to field

Cook et al. take advantage of the natural variation in cerebellar size and folding between two strains of laboratory mice to examine whether they could arise from differences in the degree of

© 2024. Published by The Company of Biologists under the terms of the Creative Commons Attribution License (https://creativecommons.org/licenses/by/4.0/).

expansion of the EGL over the cerebellar core during development. The approach is straightforward and observational/semi-quantitative, building on the key finding that individual lobules scale independently between the two species.

Comments for the author

This starts as a unique and beautifully done study that I REALLY want to see published. It's a fundamental theme, it's a simple and basic question, it's a beautiful experimental approach and design – BUT I find that unfortunately, this paper suffers from a lack of rigor in the presentation, including loose use of language; grossly insufficient figure legends; and most importantly, a lack of mathematical treatment. I tried to be as thorough as possible to be helpful to the authors, but I must apologize: around line 400, the lack of mathematical treatment made going on with this review pointless, because all the really interesting findings regarding cell division angles must be crossed with a solid description then understanding of how folding differs between the two strains – but that description is presently semi-quantitative at best, so no synthesis of the findings is currently possible.

The good news is that all of that is easily fixable. My impression is that the authors haven't realized that they have in hands everything they need to test the mathematical predictions of the model presented by Mota and Herculano-Houzel (2015) regarding how folding depends on the relative expansion of the surface area compared to the thickness of a growing cortex. From the graphs presented, it seems to me that they are making exactly the predicted case: that whatever causes a developing cortex to expand faster in surface area relative to thickness will make it fold more, and by predictable amounts. This point is made specifically for the cerebellum in York et al., available in bioRxiv, but it already follows from Mota and Herculano-Houzel (2015).

I apologize for taking so long to finally be able to review this paper, only to then abandon the reading, but I mean to be helpful to the authors and save them time. The work is beautifully done, but I find the treatment of the results to be subpar and not worthy of publication yet. I would be delighted to review this paper again (immediately, I promise!) once the authors can give their data proper mathematical treatment to test the hypotheses stated in the model of Mota and Herculano-Houzel (2015). They can use the same equations, although the exponent will be different for 2D sections (see supplemental material). Specifically: please test the prediction that BOTH strains should STILL fit the same scaling relationship, which would indicate that what differs between the two strains is the expansion in surface area relative to the expansion in thickness of the EGL as the cerebellar cortex develops. I believe that testing this hypothesis would greatly simplify the interpretation and discussion of the findings.

I have also listed below under "major points" a number of issues that require more precise definitions or careful wording, or better documentation of the results.

Huge points The summary statement uses a common formulation that is however problematic for it sets the cerebellum as an agent of its own morphogenesis ("the cerebellum adjusts… to regulate…). I suggest rewriting to something like "Cell division angle within the external granule layer varies regionally with consequences for the tissue mechanics that sets the amount and wavelength of cortical folding".

Overall, this manuscript is lacking in formality. The use of language in the description of the variables is very loose, which detracts from its readability and the interpretation of the data. For example, the key variables are never defined, and the wording used is not precise enough to specify what is being referred to (see below in minor points). The paper also makes indiscriminate and improper use of the words "rate" and "ratio". The former implies change over time, which is not what the graphs depict; the latter refers explicitly to a division, which is implied by the SLOPE of said graphs, but is not what is plotted or measured. Please review the entire paper to correct the language. Please see suggestions below in Minor points.

Similarly, the expressions "balanced-expansion" and "tissue geometry" are recurring, but they are never properly defined, so their meaning is vague and the reader worries about circularity.

© 2024. Published by The Company of Biologists under the terms of the Creative Commons Attribution License (https://creativecommons.org/licenses/by/4.0/).

It gets worse when these two problems are combined, as in lines 253-254: “in both strains the growth ratio of L8 closely approximates the geometrically determined balanced-expansion profile during the critical period”. I understand all the words, but I honestly cannot start to imagine what the authors mean. It goes on in lines 255-256: “while the growth ratios between L4-5, L6-7, and L8 are similar within each strain…” – what is meant by growth ratios BETWEEN lobules? Do the authors mean that the midsagittal perimeter of all three lobules expands as similar functions of midsagittal surface area both within each strain (that is, a shared function across all three lobules) and across the two strains? Please use clearer, more specific language to help your readers understand what is on your mind…

The Results section as a whole left me thinking that this paper currently lacks a proper mathematical treatment of this BEAUTIFUL dataset, which is a huge missed opportunity to test the predictions of Mota and Herculano-Houzel (2015) regarding scaling. From the figures and data points (see below), I suspect that FVB/NJ has an EGL that expands much more quickly in (pial) surface area than in (radial) thickness compared to C57Bl/6J – which is exactly the condition that Mota and Herculano-Houzel (2015) predict that will lead to increased folding. The authors have also missed the opportunity to do this analysis lobe by lobe, with the invaluable internal controls of those lobules that DON’T differ in folding between the two strains. I strongly urge the authors to look up the rather simple predictive equations in Mota and Herculano-Houzel (2015) and test them using the present data. All that they are missing to do that is a direct measurement of the thickness of the expanding EGL – which for now can be approximated by the ratio between midsagittal surface area and midsagittal total perimeter of the pial surface.

Major points line 57 – do the authors really mean that the EGL expands only along the A-P axis, not M-L? Is the migration of the daughter cells that specific? Please elaborate, as this preferential A-P migration is probably fundamental for explaining the accordion-like 2D folding of the cerebellar surface, unlike the expansion and folding of the cerebral cortex. Could it be that the A-P expansion is the only one that is captured by analysis restricted to the midsagittal plane? How does the cerebellum expand in the M-L plane, then?

Line 64-65 – “how the geometry of the unfolded tissue sets the ratio of growth”. This is obscure to the uninitiated reader. What exactly is meant by geometry here? Also, the differential expansion certainly “results” in folding; whether it is “needed for” is something else. I realize that I may be picky about this language, but I think it is an important distinction.

Line 72 – what exactly is meant by “wavelength of folding”? Please be precise. Is this the foliar interval? The exposed width of each folia? The PERIMETER of folia, from fundus to fundus, along the midsagittal surface?

Line 74 – the amount of folding in the cerebral cortex does not correlate with its thickness; it depends on the combination of surface area and thickness.

Lines 104-105 – the variables cross-sectional area, surface length, and positive curvature must be described in the Results (ARE THEY EVEN DESCRIBED IN THE METHODS?). Judging from the insets in Figure 1 (which are not described in the figure legend!), it seems that “cross-sectional area” is the midsagittal area of the cerebellar cortex; “surface length” is the total perimeter of the midsagittal cerebellar surface; and “folding index” (line 114) is the ratio between the total perimeter and the outer (exposed) perimeter of the midsagittal cerebellar surface. As to the “positive curvature” shown in Figure S2A, I have no idea what that is, and the figure legend is unhelpful.

Lines 107-109 – There is no “decrease” or “increase” here (because one strain cannot be established as a reference for the other, unless it is known that C57Bl/6J was DERIVED from FVB/NJ – in which case that must be mentioned in the text!), but there certainly is a difference in size. Please rephrase accurately to state that some lobules have increased midsagittal perimeter and others, decreased midsagittal perimeter. Please correct the language in the remainder of the results to reflect that C57Bl/6J is not “reduced”, but rather “smaller than”, FVB/NJ.

Lines 110-112 – expressing the perimeter of each lobule as a function of the TOTAL length is interesting but contaminated by the other lobules. I recommend plotting an additional small graph in figure 1 to show the RATIO between the perimeter of each lobule in one species compared to the

© 2024. Published by The Company of Biologists under the terms of the Creative Commons Attribution License (https://creativecommons.org/licenses/by/4.0/).

other. From eyeballing figure 1E, this additional figure will show very clearly that each lobule scales independently from the others (and L10 very little, and L8 not at all), which is a very cool finding to document.

Lines 111-113 – here is the problem with using the total midsagittal perimeter to normalize the lobules: it makes it look like some lobules that are larger in FVB/NJ are actually proportionately smaller compared to C57Bl/6J. The uninitiated reader will think that your report is contradictory, when it's really not.

Line 114 – what is positive curvature, and why use it to express the folding index? The insets in figure 1F-G imply that folding index was measured as the standard in the field: the ratio between total perimeter and exposed perimeter, not the formula given in line 114. Please clarify.

Lines 115-122 – this is a convoluted analysis that seems to point to the folding changing independently across lobules. I suggest simplifying this analysis in the following manner: (1) calculate the folding index = total lobule perimeter/exposed lobule perimeter for each lobule in each strain, as in figure 1E; (2) calculate the RATIO in folding index for each lobule between the two strains, as suggested above (lines 110-112); then (3) TEST the prediction that there is a DIRECT CORRELATION between how much the folding of each lobule differs and how much its perimeter differs between the two strains. From eyeballing figure 1, it looks like lobules 4/5 and 9 are also more folded in FVB/NJ than C57Bl/6J, so it is a really important question to determine whether that difference is proportional to the simple difference in perimeter (or, rather, the ratio between perimeter and thickness; see York et al. in bioRxiv, https://doi.org/10.1101/2023.05.17.541232). Importantly, the result remains that there is a robust difference in folding between the two strains in L6-7, which does provide a tractable system for what the authors intend to test.

Line 130 – why not say simply "while the adult cerebellum is three-layered and has been treated as a three-layered system also during development"? Excellent point about the developing cerebellum at this point being a 2-layered system of proliferating EGL and inner core. But please help the reader: does this mean then that at this point the Purkinje cells already exist, and form the surface of the "core"?

Lines 138-139 – This sentence is inaccurate. Figure 2A shows the growth in total midsagittal perimeter as a function of growth in total midsagittal area. Now, the interesting thing that the authors seem to be overlooking is that the RATIO between the midsagittal surface area and the perimeter is a rough estimate of the THICKNESS of the developing cortex. That the two increase hand in hand up to a certain point in both strains means exactly that growth is isometric until then, that is, without changes in shape; at a certain point, perimeter starts to increase faster than area in FVB/NJ than in C57Bl/6J – and this is probably when folding begins.

Lines 153-155 – Please rewrite to clarify, the sentence is ambiguous in many ways. "Early" needs to be specified: it is defined exactly as that part of the curves that overlap between the strains. The important finding to me is that the two strains START development similarly, and THEN they diverge – which prompts the following question of HOW they start to diverge.

Lines 158-163 – this is indeed a very tempting interpretation, BUT inspection of figure 2A-B with a ruler shows that FVB/NJ is already MORE folded than C57Bl/6J well BEFORE the points when midsagittal perimeter x midsagittal area start to diverge. Indeed, it seems that applies from the very beginning. How do the authors explain that?

Lines 182 and 187 – What exactly is meant by "geometry", here and throughout the paper? In line 182, geometry seems to refer to the expansion in midsagittal perimeter relative to midsagittal area; but in line 187, geometry seems to refer rather to shape and position of… something that might determine where the folds form. Please clarify, and maybe use a different word other than "geometry".

Lines 192-193 – here is another problem, this time with another, implied, meaning of "geometry": the positioning of the anchoring centers. Also: isn't every new pit of a fold a new anchoring center?

© 2024. Published by The Company of Biologists under the terms of the Creative Commons Attribution License (https://creativecommons.org/licenses/by/4.0/).

Lines 196-200 – again the problem with the use of “rates” and “ratios”. Figure 3 doesn’t show ratios explicitly; and rates imply time, but the x axis is not time. What is being analyzed here is the relative increase in midsagittal perimeter over midsagittal surface area – which makes me think that what is REALLY going on that is relevant is that for a similar midsagittal perimeter, FVB/NJ have THINNER cerebellar cortices – which is exactly the condition that Mota and Herculano-Houzel (2015) predicted that would lead to increased folding. (Note: please correct “predicted” in Figure 3B, it’s missing the c).

Paragraph starting at line 213 – I can’t really understand what is being proposed here. “Tissue geometry” was never defined, and my concern is that the argument is actually circular. I also don’t understand the concept that with “balanced-expansion” no folding will occur. Please define “balanced-expansion”. I wonder if what is missing here isn’t proper mathematical treatment of the data, for instance testing the predictions of the model put forth in Mota and Herculano-Houzel (2015).

Line 259 – isn’t the “complex geometry” exactly the pattern of fissuring, so isn’t the onset of new fissures exactly what makes a more complex geometry? Please clarify. What is the complex geometry? Is “geometry” meant to signify shape?

Lines 262-264 – It seems that “greater wavelength” translates to “less folding”, which makes this qualitative statement akin to Mota and Herculano-Houzel’s quantitative, and thus testable, prediction that for a similar surface area, thicker cortices will be less folded. Please test that prediction directly, you have the data! That would transform this entire section in the paper from a qualitative into a quantitative study, which would be much more powerful. Also: please define the use of “wavelength” in this paper.

I apologize, but I stopped reading around line 400. Everything else from here on hinges on the analysis of the previous data, which I find sorely lacking in mathematical treatment that would be so, so simple to carry out.

Minor points

line 52 – arranged, not arraigned

line 54 – only the second phase of cerebellar development is mentioned; what is the first phase?

Rebuttal letter

Dear Dr Silver,

We appreciate your work in finding and handling the reviewers as well as the very thorough and positive reviews we received of our manuscript entitled “Cell division angle regulates the tissue mechanics and tunes the amount of cerebellar folding”. We appreciate that our work was recognized to be “an important aspect of cerebellar development” and that it is a “unique and beautifully done study.” We also appreciate the comment that “This is an exceptionally well-written manuscript.”

Given the positive reviews and the lack of requests for additional experimental data we were surprised to have the paper rejected. We think the reviewer requests are less extensive than may first appear. And we believe that we can satisfactorily address all the comments by both reviewers in a timely manner by adding additional analysis of our data set and adding the results of one new experiment, as summarized here and with additional details provided below in a response to the reviewers’ comments.

Reviewer 1

© 2024. Published by The Company of Biologists under the terms of the Creative Commons Attribution License (https://creativecommons.org/licenses/by/4.0/).

They requested 4 areas of clarification. We believe we can address two of their requests for clarification with edits to the text. Secondly, we can provide the results of a new experiment that will go beyond their request for clarification and experimentally address the other two points raised.

Reviewer 2
They listed 27 issues to address. **Of the 27 issues, 21 were repeated requests for textual clarity of the same repeated words**. They also made **5 repeated requests** that we analyze our data set using the equation provide in Mota and Herculano-Houzel (2015). We have now mathematically treated our developmental data as they did in Mota and Herculano-Houzel (2015) and can supply this new analysis (See attached figure). The basic finding is that our data comparing two inbred strains of mice do not support the 2015 prediction based on comparing different species that folding amount is set during development by one universal scaling relationship between the exposed surface area and the thickness and surface area of the cortex. Rather, our results indicate that during development the strains show unique relationships between these measures. This result is in line with our findings that the tangential expansion of the EGL and its thickness are regulated during development to give rise to the folding amounts seen within the adult tissue. Thus, several factors contribute to folding amount, rather than there being a single scaling relationship.

Therefore, we would like to ask that you consider changing your decision and allow us to submit a revision instead of a new submission. If you are not able to make that adjustment, then we would consider resubmitting a new paper to *Development* if you commit to asking the same two reviewers to review our improved manuscript a second time. Both reviewers indicated they would like to, and even expected to, review a revised version. We understand that it is nevertheless possible one would refuse, and if so, you might then feel compelled to invite a new reviewer.

Please find a synopsis of how we would respond to each of the reviewer's comments below as well as a numbered and color-coded list of Reviewer 2's full comments.

Thank you again for your work in finding these reviewers and corralling them over the summer. We look forward to your response.

Sincerely,

Andrew K Lawton

Synopsis of response plan to Reviewer 1.

1. We believe we can clarify points **1** and **4** through appropriate textual changes.

- They ask "…Does the composition of the EGL (proliferating vs differentiating cells) matter or is it entirely based on thickness irrespective of whether there is greater proliferation vs differentiation?"
    - This is an interesting question. We will take this opportunity to better describe the EGL and explain that as granule cells mature they move from the outer EGL (oEGL) to the inner (iEGL) and then orient their cell body and begin extending their processes in the medial lateral orientation. It is therefore plausible that the differences in cell orientation and fiber density between the layers could play a part, but analysis of this is beyond the scope of the current study.
- They state, "The authors surmise that a higher density of Purkinje cells in FVB could result in an increased secretion of mitogen, Sonic hedgehog. Considering that there is no difference in proliferation levels between the two strains how would this be likely? This sentence should either be excluded or an ISH for Shh or any readouts of the Shh pathway need to be included to prove their point."
    - For reference the Current Text Line 362-366: As the Purkinje cells eventually form a single monolayer throughout the cerebellum, the higher density in FVB/NJ during the critical period of L6-7 could indicate that the regional density of Purkinje cells, and by extension the amount of SHH secreted, may prefigure the final amount of folding through increasing the expansion of the EGL.

© 2024. Published by The Company of Biologists under the terms of the Creative Commons Attribution License (https://creativecommons.org/licenses/by/4.0/).

- o We agree that at this point in the results, especially prior to the proliferation data, we should not speculate on the various possible molecular mechanisms linking the cellular differences possibly prefiguring the folding amount. Further, in the discussion we already speculate how both the number of Purkinje cells, as well as any strain intrinsic differences in effectiveness of Purkinje cells may both have roles in setting folding amount. We will be sure this point is clear in the Discussion.

2. We believe we can address points **2** and **3** through an additional experiment, both of which are fascinating questions.

- They state, “Since plane of cell division seems to be the major determining factor of EGL thickness, does plane of division have any influence on subsequent cell fate (renewal vs differentiation) between the two strains? In the cerebral cortex, vertical divisions are often associated with self-renewal whereas horizontal divisions are often asymmetric.”
  - o We are only aware of one paper by Rosalind Segal’s lab that described a role for SHH in symmetrical cell divisions of GCPs (Merk et al, Dev Neurosci, 2020). However, the live imaging to track cell division outcome was performed in slice culture, where we know GCP behaviors are altered compared to in vivo because cells migrate away from the surface as well as down the Bergmann glia into the IGL. More importantly, several clonal analysis studies of GCPs marked in the embryo or soon after birth demonstrated that GCPs divide only symmetrically, first to expand the GCP pool through producing two GCPs at each cell division, and then in the final division to produce two post mitotic GCs (Espinosa and Luo, J. Neurosci., 2008; Legué et al, Development 2015; Zong et al., Cell 2005). We will add this important information to the Introduction.
- They state, “Could there be migratory differences between the two strains, with cellular movement being in the direction of oEGL to inner EGL within C57, while FVB exhibiting more tangential movement?”
  - o Given the symmetric mode of GCP division, migration to the iEGL basically means the cell has adopted a differentiation fate. Thus, we think points 2 and 3 are asking the same question, is there more (or less) differentiation in C57 mice compared to FVB.
  - o A full investigation into how cell division angle affects thickness is itself another project. Similarly, detailing the cell-movement mechanics in each strain would require placing inducible CRE alleles and reporter alleles into both FVB/NJ and C57Bl/6J backgrounds requiring many generations of breeding back to each inbred line, which is beyond the scope of this project.
  - o However, we believe we can address the general question of whether the propensity of GCPs to differentiate is different between the strains using a differentiation assay. We will mark proliferative granule neurons in the oEGL with EDU and measure the percentage of labeled cells that arrive in the iEGL after 24 hours.
  - o We would predict that, if cell-division angle influences cell fate, the cell division angle would also predict the percentage of EDU labeled cells that move to the iEGL during the chase period. For example, if there are differences in cell differentiation/motility such that B6 has more differentiation and thus migration into the iEGL we predict that more EDU+ cells will be found within the iEGL of C57Bl/6J mice after 24 hr.

Synopsis of Response plan to Reviewer 2:
We have numbered the comments made by reviewer 2 to make it simpler for us to address ones that are repetitive. We believe we can address each comment of reviewer two in a timely manner by textual clarification and by supplying the new data analysis requested.

- They posed **21** repeated requests for textual clarifications of two important points (1-4, 6-8, 10, 11, 14, 16-18, 20-27). See Cyan Highlight below
  - o Change our use of “rate” and “ratio” to be more consistent and correct (points: 2,4, 17, 22). We thank the reviewer for pointing this out and will make the changes.

© 2024. Published by The Company of Biologists under the terms of the Creative Commons Attribution License (https://creativecommons.org/licenses/by/4.0/).

  - Better define the words: “geometry,” “wavelength,” and “balanced expansion” (points: 3, 4, 7, 20, 21, 23, 24, 25). Again, we thank the reviewer for pointing out this import clarification and will make the changes.
  - We also point out that perhaps the reviewer missed some of our descriptions.
    - For Instance, we defined “wavelength” at line 292 as “the direct distance between the anchoring centers” and we also provided a visual depiction of the wavelength measured in Figure 4G.

- They posed repeated requests (4) that we analyze our existing data in the manner published by Mota and Herculano-Houzel as a “mathematical treatment.” (points: 5, 22, 23, 25) See Yellow Highlight below
  - There are numerous different models, predictions, and simulations that have been published based on adult brain tissue of what might be controlling the amount of brain folding, including the 2015 paper refenced by reviewer two.
  - Our goal here is to use a developmental investigation to determine which, if any, of these predicted tissue mechanics are present (actually occurring) during development.
    - We argue that our work provides evidence of the developmental mechanics *that give rise to* the geometric relationships observed in the adult brain that were reported in Mota and Herculano-Houzel 2015.
    - Therefore, we believe our work is building upon and going beyond the work of a variety of predictive modeling based on adults, including the referenced 2015 paper.
  - We have run our developmental data set through the mathematical treatment of the 2015 paper.
    - We used the reviewer’s recommendation (Point 5) to calculate the thickness as the “ratio between midsagittal surface area and midsagittal total perimeter of the pial surface.”
    - *Please see the attached plots, the residuals, and the fitting parameters that resulted from this treatment.*
    - In Mota and Herrculano-Houzel 2015 the authors predict that the cerebral cortex, from any species no matter how folded, should have the same relationship between the exposed surface area (an indication of folding amount) and the thickness and surface area of the cortex. The authors argue that this geometric relationship “applies across cortical development.” Therefore, throughout development the two mouse strains should have the same uniform relationship between the exposed surface area and the thickness and surface area of the cortex no matter their level of folding. Plotting as requested should show that the strains are indistinguishable from each other and that both strains can be well approximated by one combined fitting.
    - The data show that while the two strains are similar, they are clearly distinguishable. As the exposed surface increases FVB/NJ trends above the fit line and C57Bl/6J falls below the fit line (A).
    - *Science* has appended two technical papers to the Mota and Herculano-Houzel 2015 paper. In accordance with their critiques of the 2015 paper we have 1) avoided log-log plotting which minimizes the deviations present and 2) provided the residuals of the fitting so that the goodness of fit may be analyzed
    - Our data show that the residuals are not uniform, or evenly dispersed, along the length of the combined fit (C). Rather the points oscillate above and below the fitting curve. Further, the data also show that C57Bl/6J and FVB/NJ diverge as folding progresses (C). This argues that the universal fitting is not ideal as the strains are behaving differently during development.
    - Additionally, we provide analysis where each strain is treated separately (B). In this treatment, the residuals are not as large, and are more uniformly dispersed along the length of the fit indicating an improved goodness of fit (C). We also provided the fitting parameters and their 95% confidence intervals (D) in line with the rest of our analysis.
    - These results show that there is not one universal relationship between these measures in the two strains and therefore we argue that the data from cerebellum development does not support the 2015 model that predicts folding amount is set through a single universal relationship between the exposed

© 2024. Published by The Company of Biologists under the terms of the Creative Commons Attribution License (https://creativecommons.org/licenses/by/4.0/).

surface area and the thickness and surface area of the cerebral cortex. We will discuss this finding in the Discussion.

- Point 5: They ask for data that we already provided. See Grey Highlight below.
  - The reviewer hypothesizes: “I suspect that FVB/NJ has an EGL that expands much more quickly in (pial) surface area than in (radial) thickness compared to C57Bl/6J.”
    - We measured and reported this:
      - Figure 2A
      - Figure 3A, C, D,
      - Supplemental Figure 3I,
      - Supplemental Figure 4A-D.
      - These results also were incorporated into the Model (Figure 7)
  - The reviewer asks us to measure the thickness of the EGL. We had measured and reported the thickness of the EGL in:
    - Figure 4A-F
    - Figure 6I
    - Supplemental Figure 6A.
    - EGL thickness was incorporated in the Model (Figure 7).
- In Point 17 (see red highlight) the reviewer seems to mistakenly state that the “hand in hand” expansion in Figure 2A indicates Isometric scaling and that no shape changes should result.
  - Isometric scaling of a 2-D object does not produce a “hand in hand” expansion.
  - Plotting the length/area of objects under Isometric scaling produces power curves. See Supplemental Figure 4F.
  - We demonstrate that the linear-like relationship of the expansion seen in Figure 2A is not isometric but differential. We also demonstrate in Figure 2B that during this period of growth the shape of the cerebellum changes.

Numbered list of Reviewer Two’s Points:
Cyan – Request for textual clarification
Yellow – Request for treating data with Mota (2015)
Grey – Requests for data already supplied.
Red – Incorrect statements

1) The summary statement uses a common formulation that is however problematic for it sets the cerebellum as an agent of its own morphogenesis (“the cerebellum adjusts… to regulate…). I suggest rewriting to something like “Cell division angle within the external granule layer varies regionally with consequences for the tissue mechanics that sets the amount and wavelength of cortical folding”.
2) Overall, this manuscript is lacking in formality. The use of language in the description of the variables is very loose, which detracts from its readability and the interpretation of the data. For example, the key variables are never defined, and the wording used is not precise enough to specify what is being referred to (see below in minor points). The paper also makes indiscriminate and improper use of the words “rate” and “ratio”. The former implies change over time, which is not what the graphs depict; the latter refers explicitly to a division, which is implied by the SLOPE of said graphs, but is not what is plotted or measured. Please review the entire paper to correct the language. Please see suggestions below in Minor points.
3) Similarly, the expressions “balanced-expansion” and “tissue geometry” are recurring, but they are never properly defined, so their meaning is vague and the reader worries about circularity.
4) It gets worse when these two problems are combined, as in lines 253-254: “in both strains the growth ratio of L8 closely approximates the geometrically determined balanced-expansion profile during the critical period”. I understand all the words, but I honestly cannot start to imagine what the authors mean. It goes on in lines 255-256: “while the growth ratios between L4-5, L6-7, and L8 are similar within each strain…” - what is meant by growth ratios BETWEEN lobules? Do the authors mean that the midsagittal perimeter of all three lobules expands as similar functions of midsagittal surface area both within each strain (that is, a shared function across all three lobules) and across the two strains? Please use clearer, more specific language to help your readers understand what is on your mind…

© 2024. Published by The Company of Biologists under the terms of the Creative Commons Attribution License (https://creativecommons.org/licenses/by/4.0/).

5) The Results section as a whole left me thinking that this paper currently lacks a proper mathematical treatment of this BEAUTIFUL dataset, which is a huge missed opportunity to test the predictions of Mota and Herculano-Houzel (2015) regarding scaling. From the figures and data points (see below), I suspect that FVB/NJ has an EGL that expands much more quickly in (pial) surface area than in (radial) thickness compared to C57Bl/6J - which is exactly the condition that Mota and Herculano-Houzel (2015) predict that will lead to increased folding. The authors have also missed the opportunity to do this analysis lobe by lobe, with the invaluable internal controls of those lobules that DON'T differ in folding between the two strains. I strongly urge the authors to look up the rather simple predictive equations in Mota and Herculano-Houzel (2015) and test them using the present data. All that they are missing to do that is a direct measurement of the thickness of the expanding EGL - which for now can be approximated by the ratio between midsagittal surface area and midsagittal total perimeter of the pial surface.
6) line 57 - do the authors really mean that the EGL expands only along the A-P axis, not M-L? Is the migration of the daughter cells that specific? Please elaborate, as this preferential A-P migration is probably fundamental for explaining the accordion-like 2D folding of the cerebellar surface, unlike the expansion and folding of the cerebral cortex. Could it be that the A-P expansion is the only one that is captured by analysis restricted to the midsagittal plane? How does the cerebellum expand in the M-L plane, then?
7) Line 64-65 - "how the geometry of the unfolded tissue sets the ratio of growth". This is obscure to the uninitiated reader. What exactly is meant by geometry here? Also, the differential expansion certainly "results" in folding; whether it is "needed for" is something else. I realize that I may be picky about this language, but I think it is an important distinction.
8) Line 72 - what exactly is meant by "wavelength of folding"? Please be precise. Is this the foliar interval? The exposed width of each folia? The PERIMETER of folia, from fundus to fundus, along the midsagittal surface?
9) Line 74 - the amount of folding in the cerebral cortex does not correlate with its thickness; it depends on the combination of surface area and thickness.
10) Lines 104-105 - the variables cross-sectional area, surface length, and positive curvature must be described in the Results (ARE THEY EVEN DESCRIBED IN THE METHODS?). Judging from the insets in Figure 1 (which are not described in the figure legend!), it seems that "cross-sectional area" is the midsagittal area of the cerebellar cortex; "surface length" is the total perimeter of the midsagittal cerebellar surface; and "folding index" (line 114) is the ratio between the total perimeter and the outer (exposed) perimeter of the midsagittal cerebellar surface. As to the "positive curvature" shown in Figure S2A, I have no idea what that is, and the figure legend is unhelpful.
11) Lines 107-109 - There is no "decrease" or "increase" here (because one strain cannot be established as a reference for the other, unless it is known that C57Bl/6J was DERIVED from FVB/NJ - in which case that must be mentioned in the text!), but there certainly is a difference in size. Please rephrase accurately to state that some lobules have increased midsagittal perimeter and others, decreased midsagittal perimeter. Please correct the language in the remainder of the results to reflect that C57Bl/6J is not "reduced", but rather "smaller than", FVB/NJ.
12) Lines 110-112 - expressing the perimeter of each lobule as a function of the TOTAL length is interesting but contaminated by the other lobules. I recommend plotting an additional small graph in figure 1 to show the RATIO between the perimeter of each lobule in one species compared to the other. From eyeballing figure 1E, this additional figure will show very clearly that each lobule scales independently from the others (and L10 very little, and L8 not at all), which is a very cool finding to document.
13) Lines 111-113 - here is the problem with using the total midsagittal perimeter to normalize the lobules: it makes it look like some lobules that are larger in FVB/NJ are actually proportionately smaller compared to C57Bl/6J. The uninitiated reader will think that your report is contradictory, when it's really not.
14) Line 114 - what is positive curvature, and why use it to express the folding index? The insets in figure 1F-G imply that folding index was measured as the standard in the field: the ratio between total perimeter and exposed perimeter, not the formula given in line 114. Please clarify.
15) Lines 115-122 - this is a convoluted analysis that seems to point to the folding changing independently across lobules. I suggest simplifying this analysis in the following manner: (1)

© 2024. Published by The Company of Biologists under the terms of the Creative Commons Attribution License (https://creativecommons.org/licenses/by/4.0/).

calculate the folding index = total lobule perimeter/exposed lobule perimeter for each lobule in =each strain, as in figure 1E; (2) calculate the RATIO in folding index for each lobule between the two strains, as suggested above (lines 110-112); then (3) TEST the prediction that there is a DIRECT CORRELATION between how much the folding of each lobule differs and how much its perimeter differs between the two strains. From eyeballing figure 1, it looks like lobules 4/5 and 9 are also more folded in FVB/NJ than C57Bl/6J, so it is a really important question to determine whether that difference is proportional to the simple difference in perimeter (or, rather, the ratio between perimeter and thickness; see York et al. in bioRxiv, https://doi.org/10.1101/2023.05.17.541232). Importantly, the result remains that there is a robust difference in folding between the two strains in L6-7, which does provide a tractable system for what the authors intend to test.

16) Line 130 – why not say simply "while the adult cerebellum is three-layered and has been treated as a three-layered system also during development"? Excellent point about the developing cerebellum at this point being a 2-layered system of proliferating EGL and inner core. But please help the reader: does this mean then that at this point the Purkinje cells already exist, and form the surface of the "core"?
17) Lines 138-139 – This sentence is inaccurate. Figure 2A shows the growth in total midsagittal perimeter as a function of growth in total midsagittal area. Now, the interesting thing that the authors seem to be overlooking is that the RATIO between the midsagittal surface area and the perimeter is a rough estimate of the THICKNESS of the developing cortex. That the two increase hand in hand up to a certain point in both strains means exactly that growth is isometric until then, that is, without changes in shape; at a certain point, perimeter starts to increase faster than area in FVB/NJ than in C57Bl/6J - and this is probably when folding begins.
18) Lines 153-155 – Please rewrite to clarify, the sentence is ambiguous in many ways. "Early" needs to be specified: it is defined exactly as that part of the curves that overlap between the strains. The important finding to me is that the two strains START development similarly, and THEN they diverge – which prompts the following question of HOW they start to diverge.
19) Lines 158-163 - this is indeed a very tempting interpretation, BUT inspection of figure 2A-B with a ruler shows that FVB/NJ is already MORE folded than C57Bl/6J well BEFORE the points when midsagittal perimeter x midsagittal area start to diverge. Indeed, it seems that applies from the very beginning. How do the authors explain that?
20) Lines 182 and 187 – What exactly is meant by "geometry", here and throughout the paper? In line 182, geometry seems to refer to the expansion in midsagittal perimeter relative to midsagittal area; but in line 187, geometry seems to refer rather to shape and position of… something that might determine where the folds form. Please clarify, and maybe use a different word other than "geometry".
21) Lines 192-193 – here is another problem, this time with another, implied, meaning of "geometry": the positioning of the anchoring centers. Also: isn't every new pit of a fold a new anchoring center?
22) Lines 196-200 - again the problem with the use of "rates" and "ratios". Figure 3 doesn't show ratios explicitly; and rates imply time, but the x axis is not time. What is being analyzed here is the relative increase in midsagittal perimeter over midsagittal surface area - which makes me think that what is REALLY going on that is relevant is that for a similar midsagittal perimeter, FVB/NJ have THINNER cerebellar cortices – which is exactly the condition that Mota and Herculano-Houzel (2015) predicted that would lead to increased folding. (Note: please correct "predicted" in Figure 3B, it's missing the c).
23) Paragraph starting at line 213 – I can't really understand what is being proposed here. "Tissue geometry" was never defined, and my concern is that the argument is actually circular. I also don't understand the concept that with "balanced-expansion" no folding will occur. Please define "balanced-expansion". I wonder if what is missing here isn't proper mathematical treatment of the data, for instance testing the predictions of the model put forth in Mota and Herculano-Houzel (2015).
24) Line 259 – isn't the "complex geometry" exactly the pattern of fissuring, so isn't the onset of new fissures exactly what makes a more complex geometry? Please clarify. What is the complex geometry? Is "geometry" meant to signify shape?
25) Lines 262-264 – It seems that "greater wavelength" translates to "less folding", which makes this qualitative statement akin to Mota and Herculano-Houzel's quantitative, and thus testable, prediction that for a similar surface area, thicker cortices will be less folded.

© 2024. Published by The Company of Biologists under the terms of the Creative Commons Attribution License (https://creativecommons.org/licenses/by/4.0/).

Please test that prediction directly, you have the data! That would transform this entire section in the paper from a qualitative into a quantitative study, which would be much more powerful. Also: please define the use of “wavelength” in this paper.

26) line 52 – arranged, not arraigned
27) line 54 – only the second phase of cerebellar development is mentioned; what is the first phase?

Rebuttal decision letter

MS ID#: DEVELOP/2023/202184

MS TITLE: Cell division angle regulates the tissue mechanics and tunes the amount of cerebellar folding

AUTHORS: Amber G Cook, Taylor V Bishop, Hannah R Crowe, Daniel N Stevens, Lauren Reine, Alexandra L Joyner, and Andrew K Lawton
ARTICLE TYPE: Research Article

Thank you for your appeal on your recently rejected manuscript and for sharing your detailed rebuttal plan. I do understand your disappointment, but given the fact that reviewer 2 felt unable to comment on part of your study as written, I saw little option other than to decline the paper.

However, we are always willing to give authors the chance to defend their manuscripts, and I do recognize that you make some valid comments in your letter. The addition of new wet and dry experiments as well as clarification of text sound reasonable. Therefore we would be willing to reconsider a revised version of your manuscript that deals, as far as possible, with the points raised by the reviewers. As I wrote in my initial decision letter, I also think you need to modify the title of your study as well as some conclusions which currently imply a causal relationship which is not shown. Upon resubmission, please provide a detailed response to the reviewers' comments and highlighting particularly any concerns that have not been included in the revised manuscript.

The revised manuscript and rebuttal will be sent to the original reviewers (if they are still available). If they are convinced by your arguments, then we would be able to consider the manuscript for publication.

First revision

Author response to reviewers' comments

Reviewer 1:

In this manuscript, Cook et al, study folding and foliation in the cerebellum and determine the cellular basis of this enigmatic phenomenon. The authors test multiple predictions for neural tissue folding during cerebellar foliation including external granule layer (EGL) thickness, EGL proliferation, Purkinje cell density, and finally, plane of cell division. Based on their rigorous analyses they discover that plane of cell division is the only factor that contributes to the regulation of EGL thickness.

This is an exceptionally well-written manuscript on an important aspect of cerebellar development that has bewildered researchers in the field. The authors have carefully considered multiple possibilities and tested each one of them. While this paper would have benefitted from analysis of mice with foliation defects, I hope this is being pursued as a separate study.

While I strongly recommend this manuscript for publication, the following points need to be clarified:

© 2024. Published by The Company of Biologists under the terms of the Creative Commons Attribution License (https://creativecommons.org/licenses/by/4.0/).

We appreciate that the reviewer finds this “an exceptionally well-written manuscript” and that it is covering an “important aspect of cerebellar development that has bewildered researchers.” Below we address each of the four comments. We address both comments 2 and 3 by providing new experimental analyses.

1. For a significant portion of the manuscript, the EGL is assumed to be a single entity. Later in the manuscript, the EGL is described as having a combination of outer proliferative and inner post-mitotic cells. When the authors describe folding wavelength being regulated by adjusting EGL thickness, does the composition of the EGL (proliferating vs differentiating cells) matter or is it entirely based on thickness irrespective of whether there is greater proliferation vs differentiation?

We thank the reviewer for the important and interesting question. We have now introduced the outer EGL (oEGL) and inner EGL (iEGL) in the introduction. As granule cells mature, they move from the oEGL to the iEGL, then orient their cell body and begin extending their processes in the medial- lateral orientation. It is therefore plausible that the differences in cell orientation and fiber density between the layers could play a part, but analysis of this is beyond the scope of the current study. It is also worth noting that the oEGL/EGL ratio is the same between the strains. Please see changes to Lines 56-58.

2. Since plane of cell division seems to be the major determining factor of EGL thickness, does plane of division have any influence on subsequent cell fate (renewal vs differentiation) between the two strains? In the cerebral cortex, vertical divisions are often associated with self-renewal whereas horizontal divisions are often asymmetric.

We again thank the review for the insightful question. Several clonal analysis studies of GCPs marked in the embryo or soon after birth demonstrated that GCPs divide only symmetrically, first to expand the GCP pool through producing two GCPs at each cell division, and then in the final division to produce two post mitotic GCs (Espinosa and Luo, J. Neurosci., 2008; Legué et al, Development 2015; Zong et al., Cell 2005). Given the symmetric mode of GCP division, cell *migration* to the iEGL basically means the cell has adopted a differentiation fate. Thus, we think points 2 and 3 ask the same question, namely, is there more (or less) differentiation in C57Bl/6J mice compared to FVB/NJ.

To address points 2 and 3 we have included new experiments:

First, we measured the rate of differentiation within the EGL during the critical period for Lobule 6/7. (Fig 5 M, O). We marked proliferative granule cells in the oEGL with a 1 hr pulse of EDU and measured the percentage of labeled cells that arrive in the iEGL after 24 hours. We found that FVB/NJ mice have a significant although only slightly lower rate of differentiation in Lobule 6/7 when compared with C57Bl/6, but specifically at the start of the critical period and not at the end of the critical period.

Second, we measured the total number of cells in the exposed EGL of L6/7 (Figure 5Q).
We found that the number of cells in the EGL is the same in FVB/N and C57Bl/6J at the start and at the end of the critical period. This result supports the conclusion that the critical factor is not the number of cells (their proliferation or even their slightly different differentiation rates) but rather the arrangement of the cells into layers. The result thus provides addition evidence for our original findings that the cell division angle is likely driving the expansion and thickness of the EGL.
Please see the paragraph starting at Line 400 in the Results.
Please see the paragraph starting at Line 522 in the Discussion.

3. Could there be migratory differences between the two strains, with cellular movement being in the direction of oEGL to inner EGL within C57, while FVB exhibiting more tangential movement?

A full detailing of the cell-movement mechanics in each strain would require placing inducible CRE alleles and reporter alleles into both FVB/NJ and C57Bl/6J backgrounds requiring many generations of breeding back to each inbred line, which is beyond the scope of this project. However, we believe we have addressed this core of this question in our response to point 2.

© 2024. Published by The Company of Biologists under the terms of the Creative Commons Attribution License (https://creativecommons.org/licenses/by/4.0/).

4. The authors surmise that a higher density of Purkinje cells in FVB could result in an increased secretion of mitogen, Sonic hedgehog. Considering that there is no difference in proliferation levels between the two strains how would this be likely? This sentence should either be excluded or an ISH for Shh or any readouts of the Shh pathway need to be included to prove their point.

> This sentence has been removed from the revised paper. We agree that at this point in the results, especially prior to presenting the proliferation data, we should not speculate on the various possible molecular mechanisms linking the cellular differences possibly prefiguring the folding amount.
> Further, in the discussion we already speculated how both the number of Purkinje cells, as well as any strain intrinsic differences in effectiveness of Purkinje cells may both have roles in setting folding amount.

Reviewer 2 Comments for the Author:
This starts as a unique and beautifully done study that I REALLY want to see published. It's a fundamental theme, it's a simple and basic question, it's a beautiful experimental approach and design – BUT I find that unfortunately, this paper suffers from a lack of rigor in the presentation, including loose use of language; grossly insufficient figure legends; and most importantly, a lack of mathematical treatment. I tried to be as thorough as possible to be helpful to the authors, but I must apologize: around line 400, the lack of mathematical treatment made going on with this review pointless, because all the really interesting findings regarding cell division angles must be crossed with a solid description then understanding of how folding differs between the two strains – but that description is presently semi-quantitative at best, so no synthesis of the findings is currently possible.

The good news is that all of that is easily fixable. My impression is that the authors haven't realized that they have in hands everything they need to test the mathematical predictions of the model presented by Mota and Herculano-Houzel (2015) regarding how folding depends on the relative expansion of the surface area compared to the thickness of a growing cortex. From the graphs presented, it seems to me that they are making exactly the predicted case: that whatever causes a developing cortex to expand faster in surface area relative to thickness will make it fold more, and by predictable amounts. This point is made specifically for the cerebellum in York et al., available in bioRxiv, but it already follows from Mota and Herculano-Houzel (2015).

I apologize for taking so long to finally be able to review this paper, only to then abandon the reading, but I mean to be helpful to the authors and save them time. The work is beautifully done, but I find the treatment of the results to be subpar and not worthy of publication yet. I would be delighted to review this paper again (immediately, I promise!) once the authors can give their data proper mathematical treatment to test the hypotheses stated in the model of Mota and Herculano-Houzel (2015). They can use the same equations, although the exponent will be different for 2D sections (see supplemental material). Specifically: please test the prediction that BOTH strains should STILL fit the same scaling relationship, which would indicate that what differs between the two strains is the expansion in surface area relative to the expansion in thickness of the EGL as the cerebellar cortex develops. I believe that testing this hypothesis would greatly simplify the interpretation and discussion of the findings.

I have also listed below under "major points" a number of issues that require more precise definitions or careful wording, or better documentation of the results.

We appreciate that the reviewer finds this work to be "unique" and on a "fundamental theme" with a "beautiful experimental approach and design." And we thank them for their careful and close reading of the text. Below we addressed the request above that we mathematically treat our data as in Mota and Herculao-Houzel (2015) and test the predictions presented there. We also provide the additional analysis that was requested and address the requests for textual clarifications.

We have numbered the comments made by reviewer 2 to make it simpler for us to address them.
1. The summary statement uses a common formulation that is however problematic for it sets the

© 2024. Published by The Company of Biologists under the terms of the Creative Commons Attribution License (https://creativecommons.org/licenses/by/4.0/).

cerebellum as an agent of its own morphogenesis ("the cerebellum adjusts… to regulate…). I suggest rewriting to something like "Cell division angle within the external granule layer varies regionally with consequences for the tissue mechanics that sets the amount and wavelength of cortical folding".

We thank the reviewer for this recommendation and have made the suggested adjustments. Please see the Summary Statement: Lines 19-20.

2. Overall, this manuscript is lacking in formality. The use of language in the description of the variables is very loose, which detracts from its readability and the interpretation of the data. For example, the key variables are never defined, and the wording used is not precise enough to specify what is being referred to (see below in minor points). The paper also makes indiscriminate and improper use of the words "rate" and "ratio". The former implies change over time, which is not what the graphs depict; the latter refers explicitly to a division, which is implied by the SLOPE of said graphs, but is not what is plotted or measured. Please review the entire paper to correct the language. Please see suggestions below in Minor points.

We thank the reviewer for their careful reading of this manuscript. We have taken the opportunity to improve the readability of the paper. We have removed any inappropriate use of "rate" and have clarified our use of ratio across the text. We use "ratio" to refer to the slopes in our graphs as recommended by the reviewer. We have better defined "balanced-expansion," "geometry," and "wavelength of folding" in the text.

A "balanced-expansion curve" result from plotting the length and area of 2-D shape undergoing isometric scaling. See Lines 231-232 and please see Supplemental Figure 5F for several examples of isometric scaling and the resulting "balanced-expansion" curves. Throughout the text we use "geometry" as defined in Oxford Languages, "the shape and relative arrangement of the parts of something." See Lines 44-45. The Wave-length of folding is the direct distance between the base of the fissures. See line 77-78.

3. Similarly, the expressions "balanced-expansion" and "tissue geometry" are recurring, but they are never properly defined, so their meaning is vague and the reader worries about circularity.

Please see response to comment #2 where we address the definition of these terms.

4. It gets worse when these two problems are combined, as in lines 253-254: "in both strains the growth ratio of L8 closely approximates the geometrically determined balanced-expansion profile during the critical period". I understand all the words, but I honestly cannot start to imagine what the authors mean. It goes on in lines 255-256: "while the growth ratios between L4-5, L6-7, and L8 are similar within each strain…" – what is meant by growth ratios BETWEEN lobules? Do the authors mean that the midsagittal perimeter of all three lobules expands as similar functions of midsagittal surface area both within each strain (that is, a shared function across all three lobules) and across the two strains? Please use clearer, more specific language to help your readers understand what is on your mind…

We thank the reviewer for this opportunity to further clarify the text. Please see response to comment #2 for the definition of geometry and balance expansion. Please see the paragraph starting at Line 261 for changes for additional clarity.

The ratio of growth (the EGL expansion and core expansion) in each of the lobule regions is quite similar. However, because the geometry (arrangement of the parts) of each lobule is different, this similar growth ratio does not produce the same level of differential-expansion. Folding was predicted to arises by the combination of the level of differential-expansion and the starting geometry of the tissue.

5. The Results section as a whole left me thinking that this paper currently lacks a proper mathematical treatment of this BEAUTIFUL dataset, which is a huge missed opportunity to test the predictions of Mota and Herculano-Houzel (2015) regarding scaling. From the figures and data points (see below), I suspect that FVB/NJ has an EGL that expands much more quickly in (pial) surface area than in (radial) thickness compared to C57Bl/6J – which is exactly the condition that

© 2024. Published by The Company of Biologists under the terms of the Creative Commons Attribution License (https://creativecommons.org/licenses/by/4.0/).

Mota and Herculano-Houzel (2015) predict that will lead to increased folding. The authors have also missed the opportunity to do this analysis lobe by lobe, with the invaluable internal controls of those lobules that DON'T differ in folding between the two strains. I strongly urge the authors to look up the rather simple predictive equations in Mota and Herculano-Houzel (2015) and test them using the present data. All that they are missing to do that is a direct measurement of the thickness of the expanding EGL – which for now can be approximated by the ratio between midsagittal surface area and midsagittal total perimeter of the pial surface.

We appreciate that the reviewer considers our data set beautiful.

To address this comment we have now run our data sets through the same analysis as in Mota and Herculano-Houzel (2015) as requested.
Please see Supplemental Figure 3 F-K. Please see text starting at line 149 in Results. Please see paragraph starting at line 451 in Discussion

We followed the reviewer's explicit recommendation we approximated the thickness as the ratio between the midsagittal surface area and the midsagittal perimeter. *Science* has appended two technical papers to the Mota and Herculano-Houzel 2015 paper. In accordance with their critiques of the 2015 paper we have 1) avoided log-log plotting which minimizes the deviations present and 2) provided the residuals of the fitting so that the goodness of fit may be analyzed.
In Mota and Herrculano-Houzel 2015 the authors predict that the adult cerebral cortex, from any species no matter how folded, should have the same relationship between the exposed surface area (an indication of folding amount) and the thickness and surface area of the cortex. The authors argue that this geometric relationship "applies across cortical development." Therefore, throughout development the two mouse strains should have the same universal relationship between the exposed surface area and the thickness and surface area of the cortex no matter their level of folding. Plotting as requested should show that the strains are indistinguishable from each other and that both strains can be well approximated by one combined fitting. Indeed, the reviewer stated, "Specifically: please test the prediction that BOTH strains should STILL fit the same scaling relationship…"

Our results revealed that the relationship between the "cortical thickness" and the exposed surface area is unique in each strain. The data show that while the two strains are similar, they are clearly distinguishable. As the exposed surface increases FVB/NJ trends above the fit line and C57Bl/6J falls below the fit line. Our measurements during development are therefore not in line with the universal relationship predicted from adult cerebral cortical tissues in Mota and Herculano-Houzel. Our data show that the residuals are not uniform, or evenly dispersed, along the length of the combined fit. Rather the points oscillate above and below the fitting curve. Further, the data also show that C57Bl/6J and FVB/NJ diverge as folding progresses. This argues that the universal fitting is not ideal as the strains are behaving differently during development resulting in a poor goodness-of- fit.

Additionally, we provide analysis where each strain is treated separately. In this treatment, the residuals are not as large, and are more uniformly dispersed along the length of the fit indicating an improved goodness of fit. We also provided the fitting parameters and their 95% confidence intervals in line with the rest of our analysis.

Since our results do not support the predicted two strains being the same, we therefore argue that the data from cerebellum development needs to be treated with a different model. There are numerous different models, predictions, and simulations that have been published based on adult brain tissue of what might be controlling the amount of brain folding, including the referenced 2015 paper. Our goal here is to use a developmental investigation to determine which, if any, of these predicted tissue mechanics are present (actually occurring) during development. We argue that our work provides evidence of the developmental mechanics *that give rise to* the geometric relationships observed in the adult brain that were reported in Mota and Herculano-Houzel 2015.
Therefore, we believe our work is building upon and going beyond the work of a variety of predictive modeling based on adults, including the referenced 2015 paper.

© 2024. Published by The Company of Biologists under the terms of the Creative Commons Attribution License (https://creativecommons.org/licenses/by/4.0/).

6. line 57 – do the authors really mean that the EGL expands only along the A-P axis, not M-L? Is the migration of the daughter cells that specific? Please elaborate, as this preferential A-P migration is probably fundamental for explaining the accordion-like 2D folding of the cerebellar surface, unlike the expansion and folding of the cerebral cortex. Could it be that the A-P expansion is the only one that is captured by analysis restricted to the midsagittal plane? How does the cerebellum expand in the M-L plane, then?

The murine cerebellum does indeed expand primarily in the anterior-posterior axis (Legué et al, Development 2015). Folding should follow the axis of expansion and in the case of the cerebellum give rise to the aligned, "accordion-like" folding. The medial-lateral axis expands little during development. We agree that the aligned folding in the vermis of the cerebellum (and alignment of underlying forces) allows for a simpler 2-D analysis than would be possible in the cerebral cortex. We believe that the cerebellar vermis is thus an excellent model to investigate brain tissue folding.

7. Line 64-65 – "how the geometry of the unfolded tissue sets the ratio of growth". This is obscure to the uninitiated reader. What exactly is meant by geometry here? Also, the differential expansion certainly "results" in folding; whether it is "needed for" is something else. I realize that I may be picky about this language, but I think it is an important distinction.

Please see the response to comment 2 about the definition of geometry and see line 71 where we changed "needed for" to "results" as recommended.

8. Line 72 – what exactly is meant by "wavelength of folding"? Please be precise. Is this the foliar interval? The exposed width of each folia? The PERIMETER of folia, from fundus to fundus, along the midsagittal surface?

Please see response to comment 2 about definition of wavelength of folding.

9. Line 74 – the amount of folding in the cerebral cortex does not correlate with its thickness; it depends on the combination of surface area and thickness.

Please see changes to line 80 to fix this misstatement.

10. Lines 104-105 – the variables cross-sectional area, surface length, and positive curvature must be described in the Results (ARE THEY EVEN DESCRIBED IN THE METHODS?). Judging from the insets in Figure 1 (which are not described in the figure legend!), it seems that "cross-sectional area" is the midsagittal area of the cerebellar cortex; "surface length" is the total perimeter of the midsagittal cerebellar surface; and "folding index" (line 114) is the ratio between the total perimeter and the outer (exposed) perimeter of the midsagittal cerebellar surface. As to the "positive curvature" shown in Figure S2A, I have no idea what that is, and the figure legend is unhelpful.

Please see paragraph starting at line 588 where we have expanded the descriptions of these measurements in the *Methods*. Also see lines 111-113 where we have clarified our measurements in the *Results* and the *Figure legend* starting at Line 714 for changes for textual clarity.
We measured the area of the midline sagittal cross-section of the cerebellum, the pial surface length of the cerebellum near the midline, and the positive curvature of the measured pial surface length. The positive curvature is a mathematical method to create a precise and robust measure of the exposed surface. Any curve may have positive or negative curvature. Regions of positive curvature have a convex shape (top of the cerebellar lobules) while regions of negative curvature have a saddle shape (the pial surface within the base of the fissures or anchoring centers). Removing the negative curvature from the pial surface length results in convex hull. One can liken it to wrapping the cerebellum tightly in saran wrap. The saran wrap does not dip into the fissures (where there is negative curvature) but remains on the outer surface.

11. Lines 107-109 – There is no "decrease" or "increase" here (because one strain cannot be established as a reference for the other, unless it is known that C57Bl/6J was DERIVED from FVB/NJ – in which case that must be mentioned in the text!), but there certainly is a difference in size.

© 2024. Published by The Company of Biologists under the terms of the Creative Commons Attribution License (https://creativecommons.org/licenses/by/4.0/).

Please rephrase accurately to state that some lobules have increased midsagittal perimeter and others, decreased midsagittal perimeter. Please correct the language in the remainder of the results to reflect that C57Bl/6J is not “reduced”, but rather “smaller than”, FVB/NJ.

We are not sure what the reviewer is requesting saying that there is no “increase” but also requesting us to “state that some lobules have *increased* midsagittal perimeter…” However, when comparing the two strains, or comparing two timepoints we have changed the text from “reduced” to “smaller than,” “less,” or some other appropriate word over the entire text.

12. Lines 110-112 – expressing the perimeter of each lobule as a function of the TOTAL length is interesting but contaminated by the other lobules. I recommend plotting an additional small graph in figure 1 to show the RATIO between the perimeter of each lobule in one species compared to the other. From eyeballing figure 1E, this additional figure will show very clearly that each lobule scales independently from the others (and L10 very little, and L8 not at all), which is a very cool finding to document.

We appreciate that the reviewer suggests our findings are cool. To address this comment, we have now carried out the requested additional analysis between the strains of mice. See Supplemental Figure 2C. We divided the median values of the lobule lengths of C57Bl/6 by the median values of the lobule lengths of FVB/N. Individual brain comparisons are not possible as Brain # 1 in the C57Bl/6J data set is not linked to Brain #1 in the FVB/NJ data set. We believe that Figure 1E clearly shows that each lobule region is larger in FVB/NJ than in C57Bl/6J (with the exception of Lobule 8). This information is now presented again with this new requested analysis is in Supplemental Figure 2C.

In contrast, by providing the lobules lengths as a percentage of the total length we are able to highlight that different lobules have *proportionally* different contributions to each of the strain’s total size. This is not contradictory with any of our results. This demonstrates that some lobules enclose a proportionally larger or smaller region of the underlying functional circuitry in the different strains. We think this is an important issue that was not previously reported. Please see line 118-121 for changes to add further clarity.

13. Lines 111-113 – here is the problem with using the total midsagittal perimeter to normalize the lobules: it makes it look like some lobules that are larger in FVB/NJ are actually proportionately smaller compared to C57Bl/6J. The uninitiated reader will think that your report is contradictory, when it’s really not.

Please see response to comment 12.

14. Line 114 – what is positive curvature, and why use it to express the folding index? The insets in figure 1F-G imply that folding index was measured as the standard in the field: the ratio between total perimeter and exposed perimeter, not the formula given in line 114. Please clarify.

Please See response to comment 10 where we addressed the definition of our measurements. Please see *Figure legend* starting at Line 714 for changes for textual clarification.

The insets in Figure 1F-G show the pial surface length and the positive curvature. We used the positive curvature, and the equation provided in line 123 to calculate the folding index. This is a mathematically precise way to measure the folding index and have been previously used in the field to quantify folding, including cerebellar folding.

15. Lines 115-122 – this is a convoluted analysis that seems to point to the folding changing independently across lobules. I suggest simplifying this analysis in the following manner: (1) calculate the folding index = total lobule perimeter/exposed lobule perimeter for each lobule in =each strain, as in figure 1E; (2) calculate the RATIO in folding index for each lobule between the two strains, as suggested above (lines 110-112); then (3) TEST the prediction that there is a DIRECT CORRELATION between how much the folding of each lobule differs and how much its perimeter differs between the two strains. From eyeballing figure 1, it looks like lobules 4/5 and 9 are also more folded in FVB/NJ than C57Bl/6J, so it is a really important question to determine whether that difference is proportional to the simple difference in perimeter (or, rather, the ratio between

© 2024. Published by The Company of Biologists under the terms of the Creative Commons Attribution License (https://creativecommons.org/licenses/by/4.0/).

perimeter and thickness; see York et al. in bioRxiv, https://doi.org/10.1101/2023.05.17.541232). Importantly, the result remains that there is a robust difference in folding between the two strains in L6-7, which does provide a tractable system for what the authors intend to test.

To address this comment, we have carried out the requested additional analysis but have not included this analysis in the revised text for the reasons below (Please see the graphs of this ratio data at the end of this document):

As requested, we calculated the folding index, the ratio of folding index, and we "test the prediction that there is a direct correlation between how much the folding of each lobule differs and how much its perimeter differs between the two strains. (Ratio Data A-C). As the reviewer points out, York et al. reports that the length of the total perimeter of the adult cerebellum predicts 97.8% of the variation in the folding index in the adult cerebella. Our results show that there is no correlation between how much the folding of each adult lobule differs between the strains and how much its perimeter differs (Ratio Data C). However, we think this analysis is technically flawed and should not be compared with the analysis in York et al. in bioRxiv. By subdividing any folded structure into its individual components, the folding index for each individual region will always trend towards zero as the individual units are themselves not folded (Ratio Data A). The only "folding" that remains is in the regions that are not subdivide into their individual lobules in FVB/N (L1/2, L4/5, L6, L9) because there is no consistent corresponding division in those regions in C57Bl/6J to compare to. If those regions in FVB/N were separated into individual lobules, the folding index would trend towards zero as well.

This type of analysis magnifies the small variations in the folding index that arise not from folding, but from bending or bulging of the individual lobules. For instance, Lobule 6 in C57Bl/6J has no Anchoring center separating it into Lobule 6A and 6B. But internally, between Lobule 4/5 and L7 the fissure itself is curved. Because of this issue, we decided to measure the folding index in the anterior, central, and posterior regions as shown in Supplemental Figure 2D. Our results indicate that the folding changes independently across the cerebellum – as the reviewer pointed out.

Please see the paragraph starting at line 123 for changes in the text for added clarity.

16. Line 130 – why not say simply "while the adult cerebellum is three-layered and has been treated as a three-layered system also during development"? Excellent point about the developing cerebellum at this point being a 2-layered system of proliferating EGL and inner core. But please help the reader: does this mean then that at this point the Purkinje cells already exist, and form the surface of the "core"?

We thank the reviewer for pointing out the need for clarification. Please see Lines 143-144 for changes to the text for additional clarity. The medial region (vermis) of the adult cerebellum is traditionally described as being composed of several layers, the outer molecular layer, the Purkinje cell layer, the inner granule layer, and the white matter layer in the middle of each lobule. It seems to us that it is inappropriate to say that the adult cerebellum is "three-layered."

The Purkinje cells are present at the earliest stage we addressed in this project (E16.5) and are within the core of the cerebellum but intermixed with other cell types and do not form a distinct layer (with Bergmann glia and interneurons) until after birth.

17. Lines 138-139 – This sentence is inaccurate. Figure 2A shows the growth in total midsagittal perimeter as a function of growth in total midsagittal area. Now, the interesting thing that the authors seem to be overlooking is that the RATIO between the midsagittal surface area and the perimeter is a rough estimate of the THICKNESS of the developing cortex. That the two increase hand in hand up to a certain point in both strains means exactly that growth is isometric until then, that is, without changes in shape; at a certain point, perimeter starts to increase faster than area in FVB/NJ than in C57Bl/6J – and this is probably when folding begins.

Please see comment 2 concerning corrections to Rate/Ratio

© 2024. Published by The Company of Biologists under the terms of the Creative Commons Attribution License (https://creativecommons.org/licenses/by/4.0/).

We respectfully disagree: The cross-sectional area and perimeter increasing in a linear relationship does not indicate isometric growth. When plotting in a linear scale, isometric growth of any 2-D shape will always produce a power curve with the area expanding faster than the perimeter. If a linear relationship is revealed it indicates that the growth is *not* isometric. Please see Supplemental Figure 5F for several examples of isometric scaling and the resulting “balanced-expansion” curves. When a shape undergoes isometric scaling, the perimeter (a linear term) and the area (a squared term) expand in such a way that they are balanced for that 2-D shape. This is what we refer to as balanced-expansion. For a circle, a balanced expansion is defined as when the perimeter expands by 2*pi*r and the area expands by pi*r^2. Under this balanced-expansion the circle increases in size but without any shape change (folding).

We show in Figure 2B that the folding amount increases almost from the start of the data set and increases rapidly while the EGL length and cerebellar area show a linear relationship.

Please see paragraph starting at line 166 for changes to the text for additional clarity.

18. Lines 153-155 – Please rewrite to clarify, the sentence is ambiguous in many ways. “Early” needs to be specified: it is defined exactly as that part of the curves that overlap between the strains. The important finding to me is that the two strains START development similarly, and THEN they diverge – which prompts the following question of HOW they start to diverge.

Please see changes to the text: Paragraph starting at Line 183.

19. Lines 158-163 – this is indeed a very tempting interpretation, BUT inspection of figure 2A-B with a ruler shows that FVB/NJ is already MORE folded than C57Bl/6J well BEFORE the points when midsagittal perimeter x midsagittal area start to diverge. Indeed, it seems that applies from the very beginning. How do the authors explain that?

We assume the reviewer meant Line 150-156. Along the trajectory of folding shown in Figure 2B there are times when some individuals of C57Bl/6J are slightly more folded at the same size and there are times when some individuals of FVB/NJ are slightly more folded at the same size. The data does not perfectly and precisely overlap in all places. Yet, only after the critical period for each strain does the folding index measurements of each strain consistently and clearly separate. We expect some of this variation to result from the similar, yet unique expansion patterns shown in Figure 2A. The fittings in Figure 2A are different for each strain showing that the total growth trajectories are not identical. While there may be additional interesting areas to investigate in future stories, we believe the data presented here is clear and supports our hypothesis and the conclusions we make.

Please see paragraph starting at line 183 for changes to the text for additional clarification.

20. Lines 182 and 187 – what exactly is meant by “geometry”, here and throughout the paper? In line 182, geometry seems to refer to the expansion in midsagittal perimeter relative to midsagittal area; but in line 187, geometry seems to refer rather to shape and position of… something that might determine where the folds form. Please clarify, and maybe use a different word other than “geometry”.

See response to comment 2 where we addressed the definition of geometry. In this one instance we feel that it would be less precise and less clear to use a different word – With the improved definition provided to the reader at the start of the manuscript, we believe the meaning will be clear.

21. Lines 192-193 –here is another problem, this time with another, implied, meaning of “geometry”: the positioning of the anchoring centers. Also: isn’t every new pit of a fold a new anchoring center?

With respect, we do not understand this comment or how our text implies an inappropriate meaning of geometry. See paragraph starting at 209.

© 2024. Published by The Company of Biologists under the terms of the Creative Commons Attribution License (https://creativecommons.org/licenses/by/4.0/).

Anchoring centers are the names given to the bases of the fissures that separate the lobules. So as development progresses, anchoring centers form and then retain their position in space, and become the base of the growing fissure. (See Sudarov and Joyner 2007 and Szulc et al 2015)

22. Lines 196-200 – again the problem with the use of “rates” and ratios”. Figure 3 doesn’t show ratios explicitly; and rates imply time, but the x axis is not time. What is being analyzed here is the relative increase in midsagittal perimeter over midsagittal surface area – which makes me think that what is REALLY going on that is relevant is that for a similar midsagittal perimeter, FVB/NJ have THINNER cerebellar cortices – which is exactly the condition that Mota and Herculano-Houzel (2015) predicted that would lead to increased folding. (Note: please correct “predicted” in Figure 3B, it’s missing the c).

Please see response to comment 2 where we addressed rates and ratios.
Please see response to comment 5 where we provided the Mota and Herculano-Houzel analysis.
We have fixed the misspelling in Figure 3B.

23. Paragraph starting at line 213 – I can’t really understand what is being proposed here. “Tissue geometry” was never defined, and my concern is that the argument is actually circular. I also don’t understand the concept that with “balanced-expansion” no folding will occur. Please define “balanced- expansion”. I wonder if what is missing here isn’t proper mathematical treatment of the data, for instance testing the predictions of the model put forth in Mota and Herculano-Houzel (2015).

Please see response to comment 2 where we addressed the definition of geometry and balanced- expansion. We trust the provided definitions bring the needed clarity to this paragraph. See Paragraph starting at line 230.
Please see comment 5 where we provided the Mota and Herculano-Houzel analysis.
Please see comment 17 where we address why no folding occurs during a “balanced-expansion.”

24. Line 259 – isn’t the “complex geometry” exactly the pattern of fissuring, so isn’t the onset of new fissures exactly what makes a more complex geometry? Please clarify. What is the complex geometry? Is “geometry” meant to signify shape?

See response to comment 2 where we address the definition of geometry. Please see line 278 for changes to the text for additional clarity.

The “onset of new fissures” does not give Lobule 4/5 in C57Bl6J a complex geometry. This lobule region in C57Bl/6J is not separated by a fissure (Please see Supplemental Figure 6F). However, the lobule 4/5 region bulges outward at the top and is more bent than its counterpart in FVB/NJ. This shape gives rise to a slight difference in the length and positive curvature (Please see white and cyan lines in Supplemental Figure 6F) and therefore in its folding index.

25. Lines 262-264 – It seems that “greater wavelength” translates to “less folding”, which makes this qualitative statement akin to Mota and Herculano-Houzel’s quantitative, and thus testable, prediction that for a similar surface area, thicker cortices will be less folded. Please test that prediction directly, you have the data! That would transform this entire section in the paper from a qualitative into a quantitative study, which would be much more powerful. Also: please define the use of “wavelength” in this paper.

Please see response to comment 2 where we addressed the definition of “wavelength.”
Please see response to comment 5 provided the Mota and Herculano-Houzel analysis.

26. line 52 – arranged, not arraigned

Thank you, the misspelling has been corrected. See line 53.

27. line 54 – only the second phase of cerebellar development is mentioned; what is the first phase?

Please see the paragraph starting at line 51 for changes in the text for additional clarification.

© 2024. Published by The Company of Biologists under the terms of the Creative Commons Attribution License (https://creativecommons.org/licenses/by/4.0/).

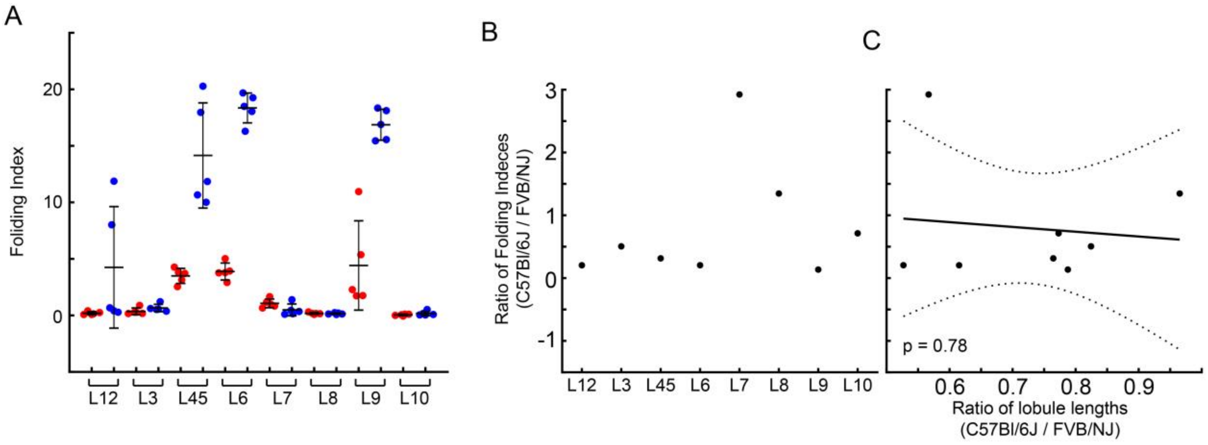

Lobule ratio data supporting (Reviewer 2: Comment 15)

Second decision letter

MS ID#: DEVELOP/2023/202184

MS TITLE: Cell division angle predicts the level of tissue mechanics that tune the amount of cerebellar folding

AUTHORS: Amber G Cook, Taylor V Bishop, Hannah R Crowe, Daniel N Stevens, Lauren Reine, Alexandra L Joyner, and Andrew K Lawton
ARTICLE TYPE: Research Article

I am happy to tell you that your manuscript has been accepted for publication in Development, pending our standard ethics checks.

Reviewer 1

Advance summary and potential significance to field

The mechanism of cerebellar foliation is a complex and highly regulated process that takes place during embryonic and early postnatal development. Foliation increases the surface area of the cerebellum, allowing for a higher density of neurons and enhancing the capabilities of the cerebellum in motor control and coordination. In this manuscript, the authors examine various hypotheses related to neural tissue folding in the process of cerebellar foliation. These hypotheses encompass factors such as the thickness of the external granule layer (EGL), EGL proliferation, Purkinje cell density, and, lastly, the orientation of cell division. Through meticulous analyses, they discover, among these factors, only the plane of cell division significantly influences the regulation of EGL thickness.

Comments for the author

The authors have responded to my feedback, and I endorse the manuscript for publication.

Reviewer 2

Advance summary and potential significance to field

The authors did a great job in revising their paper. Congratulations on a beautiful study.

© 2024. Published by The Company of Biologists under the terms of the Creative Commons Attribution License (https://creativecommons.org/licenses/by/4.0/).

Comments for the author

The authors did a great job in revising their paper. Congratulations on a beautiful study.

© 2024. Published by The Company of Biologists under the terms of the Creative Commons Attribution License (https://creativecommons.org/licenses/by/4.0/).
